# A comprehensive review of ischemic heart disease: pathophysiology, current treatments, natural products-based therapies, and nanotherapeutics

**DOI:** 10.3389/fphar.2026.1834343

**Published:** 2026-06-04

**Authors:** Amr M. Saadeldeen, Abdallah Mansour, Ahmed M. El-Dessouki, Sally A. Fahim, Abeer M. Shaheen, Rehab A. Ismail, Rania M. Salama, Riham A. El-Shiekh, Hazim O. Khalifa

**Affiliations:** 1 Department of Pharmacognosy, School of Pharmacy, Newgiza University (NGU), Giza, Egypt; 2 Pharmaceutics Department, Faculty of Pharmacy, Heliopolis University for Sustainable Development, Cairo, Egypt; 3 Pharmacology and Toxicology Department, Faculty of Pharmacy, Ahram Canadian University, Giza, Egypt; 4 Department of Biochemistry, School of Pharmacy, Newgiza University (NGU), Giza, Egypt; 5 Department of Pharmacology and Toxicology, Faculty of Pharmacy, Heliopolis University for Sustainable Development, Cairo, Egypt; 6 Clinical Pharmacy Department, School of Pharmacy, Newgiza University (NGU), Giza, Egypt; 7 Department of Pharmacognosy, Faculty of Pharmacy, Cairo University, Cairo, Egypt; 8 Department of Veterinary Medicine, College of Agriculture and Veterinary Medicine, United Arab Emirates University, Al Ain, United Arab Emirates; 9 United Arab Emirates University (UAEU) Center for Public Policy and Leadership, United Arab Emirates University, Al Ain, United Arab Emirates

**Keywords:** atherosclerosis, gut-heart axis, ischemia with non-obstructive coronary arteries, ischemic heart disease, microcirculation, phytochemicals

## Abstract

Ischemic heart disease (IHD) is one of the major cardiovascular disorders leading to global morbidity and mortality and represents a huge burden on individuals and the healthcare system worldwide. Classically, it is attributed to obstructive atherosclerotic plaques in the epicardial coronary arteries; however, it is now understood to be a heterogeneous disease that also includes microvascular dysfunction, vasospasm, and ischemia with non-obstructive coronary arteries (INOCA). IHD is triggered by modifiable and non-modifiable risk factors, and its diagnosis relies on clinical assessment, electrocardiography, cardiac biomarkers, and advanced imaging techniques. Extensive investigations and trials have established the management of IHD, including lifestyle modifications, pharmacological therapies, and revascularization, while novel interventional, regenerative, and molecularly targeted therapies are under active investigation. This review provides a comprehensive overview of IHD, integrating its epidemiology, risk factors, pathophysiology, diagnostics, and therapeutics. IHD pathogenesis is complex, involving coronary atherosclerosis, plaque disruption, thrombosis, and myocardial ischemia-reperfusion injury that are modulated by oxidative stress, inflammation, and other signaling pathways. The review also addresses the molecular mechanisms and therapeutic potential of natural bioactive compounds, including polyphenols, terpenoids, and alkaloids, which exhibit antioxidant, anti-inflammatory, and cardioprotective effects. In addition, it highlights the multifaceted nature of IHD and underscores the need for integrated, mechanism-driven approaches to improve prevention, early detection, and personalized treatment for this global health burden.

## Graphical Abstract

**Figure:**
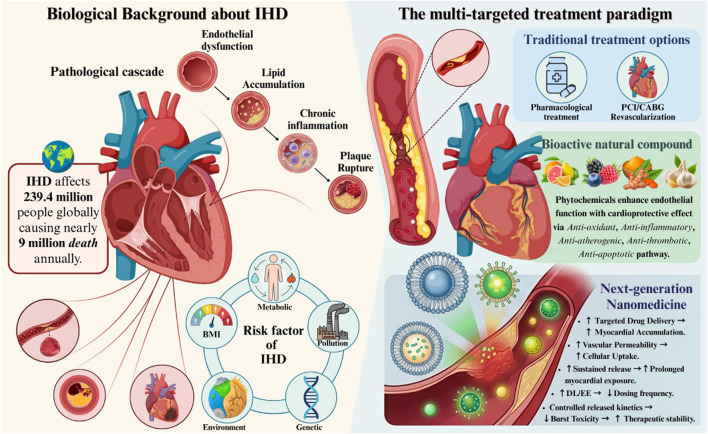

## 1 Introduction

Ischemic heart disease (IHD) refers to heart problems caused by reduced blood flow, usually due to coronary artery disease (CAD), and is a major subset of coronary heart disease (CHD) (WHO, 2017). Traditionally, IHD has been closely associated with obstructive atherosclerotic plaque formation in epicardial coronary arteries, which leads to reduced blood flow to the heart muscle (Heusch, 2019; Libby, 2021). IHD is driven by a complex interplay of modifiable and non-modifiable risk factors that have been demonstrated by extensive epidemiological research (Pencina et al., 2019). The non-modifiable risk factors include race (Howard et al., 2019), age (Hosseini et al., 2021), gender (Manfrini et al., 2020), and family history (Kim et al., 2022). On the other hand, modifiable risk factors, which encompass hypertension (Shaikh et al., 2020), hyperlipidemia (Adiputra et al., 2023), diabetes mellitus (DM) (Begum et al., 2025), and obesity (Liu S. et al., 2022). In addition to, the sedentary lifestyle factors such as alcohol consumption (Chagas et al., 2017), smoking (Salehi N. et al., 2021), physical inactivity (Winzer et al., 2018), and psychosocial stress (Helman et al., 2023) which exhibit a significant reduction in the development and progression of IHD. Notably, there are substantial sex-based differences in IHD manifestation and outcomes, with women, particularly younger women, experiencing a more complex disease spectrum influenced by female sex hormones (Gowda et al., 2024). Additionally, environmental exposure to air pollution and noise can further amplify the risk of IHD development due to mitochondrial dysfunction, thereby aggravating oxidative stress and inflammation (Montone et al., 2023; Boovarahan and Kurian, 2018).

The pathophysiology of IHD is increasingly recognized as multifaceted, involving not only large-vessel atherosclerosis but also coronary microvascular dysfunction, lipid accumulation, endothelial dysfunction, thrombosis, inflammation, and other complex mechanisms (Severino et al., 2020; Heusch, 2024). Endothelial dysfunction that occurs many years before coronary artery sclerosis is a key pathogenic hallmark in the development and progression of IHD, as it is the initial stage of an endless cycle that terminates with obvious atherosclerosis, and eventually myocardial infarction (Jerabek et al., 2020). The primary pathophysiological mechanism beyond IHD is atherosclerotic plaque formation in the coronary artery, resulting in progressive luminal narrowing, impaired myocardial perfusion, and clinical manifestations like stable angina; plaque rupture accompanied by thrombosis leading to further acute myocardial infarction (AMI) and sudden death (Stone et al., 2023). The clinical spectrum of IHD extends beyond typical obstructive coronary arteries to include conditions such as coronary microvascular dysfunction and ischemia with non-obstructive coronary arteries (INOCA), depending on the severity and duration of ischemia, which are linked to heart failure with preserved ejection fraction, recurrent angina, and lower quality of life (Mehta et al., 2022). The increasing recognition of coronary microvascular and endothelial dysfunction has highlighted the need for more advanced diagnostic and therapeutic strategies beyond conventional revascularization techniques. Accurate risk assessment, early detection, and the advancement in diagnostic methods are critical to avoiding negative outcomes, targeting the appropriate intervention, and enhancing patients’ outcomes (Lancellotti and De La Brassinne Bonardeaux, 2024). The combination of non-invasive imaging and biomarker analysis with conventional diagnostic techniques has improved accuracy in identifying patients who are at risk for IHD (Sdogkos et al., 2022). Furthermore, advancements in therapeutic approaches, including novel interventional techniques and optimized pharmacological treatments, have led to improvements in the current management of IHD (Xueying et al., 2024). Current management of IHD encompasses pharmacological therapies, lifestyle modifications, and invasive interventions such as percutaneous coronary intervention (PCI) and coronary artery bypass grafting (CABG) (Bansal and Hiwale, 2023). Recently, comprehensive treatment strategies prioritize individualized care, which targets the risk factor and the underlying pathological mechanism specific to each patient (Bertsimas et al., 2020). However, prognosis remains poor for patients with severe IHD not amenable to these therapies, underscoring the urgency of novel approaches like extracorporeal shock wave therapy to promote angiogenesis and myocardial repair. Concurrently, advances in non-invasive imaging techniques and the understanding of molecular and cellular mechanisms, including the regulatory roles of circular RNAs and stem/progenitor cell therapies, promise to improve diagnostics and therapeutic options for IHD.

Given these complexities, ongoing research is focused on delineating the diverse mechanisms underlying IHD pathophysiology and tailoring management strategies that reflect the heterogeneous nature of the disease. This review aims to comprehensively understand IHD, integrating epidemiology, risk factors, pathophysiology, diagnostic advances, and emerging therapeutic interventions. Additionally, it highlights the persisting gaps and future directions needed for improving prevention, early detection, and personalized management of this widespread condition.

## 2 The global burden of IHD: incidence, prevalence, and mortality

Cardiovascular diseases account for 32% of all global deaths, with an estimated 19.8 million deaths annually, and 85% of these deaths are attributed to IHD and stroke (WHO, 2017). According to the 2023 global burden of disease (GBD) estimates, IHD affects approximately 239.4 million people worldwide, corresponding to a prevalence of 2,968 cases per 100,000 population (≈3.1% globally). Annually, 12.7 million new cases occur, reflecting an incidence rate of 158 per 100,000 population. IHD remains a leading cause of disability and mortality, accounting for 8.9 million deaths and 192.4 million disability-adjusted life years (DALYs) across all ages (Institute for Health Metrics and Evaluation, 2023), underscoring its sustained global health and economic burden.

## 3 Life-course patterns and sex differences in IHD

Pathological changes may begin as early as the third decade of life, highlighting a concerning early onset that warrants attention, although clinical manifestations typically appear after the age of 50. Prevalence rises sharply with age, affecting ≈20% of individuals ≥70 years and 24% of those over 80 years (Hashim and Concistrè, 2023). Globally, IHD is more prevalent in males; in 2021, men accounted for 57% of cases versus 43% in women (male: female ratio ≈1.3:1) (Mahalleh et al., 2025). From age 40, men face a higher lifetime risk of developing IHD (49% vs. 32%) (Sanchis-Gomar et al., 2016). Men more commonly present with AMI, whereas women frequently develop angina or INOCA, leading to underdiagnosis. Consequently, IHD is the leading cause of female mortality worldwide, responsible for 35% of all female deaths, exceeding all cancers combined (Vogel et al., 2021).

## 4 Geographic and demographic disparities in IHD

The highest age-standardized prevalence and mortality of IHD are now observed in the Middle East and North Africa (MENA), Central Asia, and Eastern Europe, reflecting a shift away from historically dominant high-income regions (Liang et al., 2025a; Liang et al., 2025b). While high-income countries retain a substantial absolute burden due to population aging, the steepest prevalence increases occur in developing regions. China and India account for the largest absolute number of cases globally, driven by demographic growth and aging (Liang et al., 2025a). Egypt represents a critical hotspot within MENA; decomposition analyses identify Egypt, China, and other *Belt and Road* countries (China’s main international cooperation and economic strategy) as major contributors to the rising global IHD burden, largely due to population expansion and increasing metabolic risk factors, particularly hypertension and obesity (Liang et al., 2025b; Rahman, 2022).

## 5 Economic burden of IHD

The economic impact of IHD continues to escalate. In the United States, direct and indirect cardiovascular disease costs, dominated by IHD, now exceed USD 417 billion annually (Martin et al., 2025). For individuals with comorbidities, the annual direct medical cost of IHD averages 21.7% of gross domestic product (GDP) *per capita*, representing a substantial productivity loss (Shakya et al., 2025). In low- and lower-middle-income countries, this burden is often catastrophic; treatment of a single IHD episode frequently exceeds annual *per capita* public health expenditure, driving household financial collapse and straining fragile health systems (Rittiphairoj et al., 2025).

## 6 What fuels the fire: risk factors driving IHD

Classic modifiable risk factors remain central to IHD pathogenesis, primarily through mechanisms of oxidative stress, inflammation, and endothelial dysfunction key targets for both pharmacological and natural compound-based interventions. Metabolic abnormalities, including hypertension, elevated low-density lipoprotein cholesterol (LDL-c), and increased body mass index (BMI), are major contributors to disease progression and have driven a ≈67% rise in metabolic-attributed cardiovascular burden since 1990 (Shakya et al., 2025). Behavioral risks such as tobacco use and diabetes further exacerbate these pathways, particularly via chronic inflammation and vascular injury associated with nutrient-poor diets (Rittiphairoj et al., 2025).

Non-modifiable factors, including age, sex, genetics, and ethnicity, influence susceptibility by modulating these same biological processes. Aging and genetic predisposition are strongly associated with cumulative vascular damage and atherosclerosis, while sex-specific differences such as increased post-menopausal risk are partly linked to changes in hormonal regulation of inflammation and endothelial function. Ethnic disparities in IHD risk also reflect variations in metabolic and inflammatory profiles (Cheng et al., 2023; Kibel et al., 2020; Zhang and Dhalla, 2024; Severino et al., 2020).

Environmental exposures are increasingly recognized as mechanistic drivers of IHD through oxidative stress and endothelial injury. Air pollution and toxic metals such as lead promote mitochondrial dysfunction, vascular inflammation, and increased vascular resistance, reinforcing their relevance as therapeutic targets (Juricic et al., 2025; Buja and Concistrè, 2023).

**FIGURE 1:**
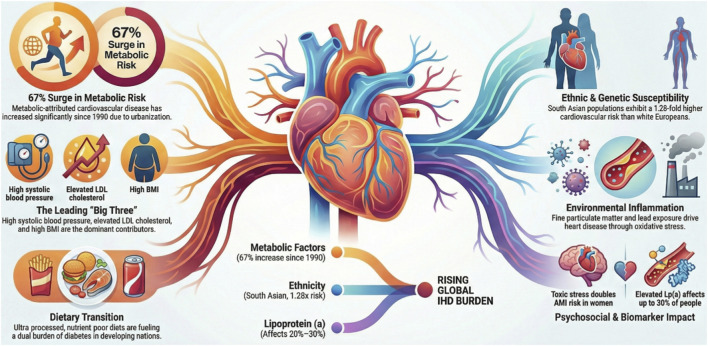
The figure depicts the interconnected drivers of the rising global IHD burden. Modifiable metabolic risk factors—particularly high systolic blood pressure, elevated LDL-c cholesterol, and high BMI—remain the dominant contributors and have fueled an estimated 67% increase in metabolic-attributable cardiovascular disease since 1990, largely driven by urbanization and dietary shifts toward ultra-processed foods. Non-modifiable determinants, including age, sex, genetics, ethnicity, and elevated lipoprotein(a), shape baseline susceptibility, with higher risk observed in South Asian populations and post-menopausal women. Environmental and psychosocial exposures—such as air pollution, lead exposure, and chronic stress—further amplify risk through inflammation, oxidative stress, and endothelial dysfunction. Together, these factors converge to drive the escalating global burden of IHD.

In addition, non-traditional risk factors, including psychosocial stress and elevated lipoprotein(a) (Lp(a)), contribute to IHD via inflammatory and pro-atherogenic pathways. Chronic stress enhances endothelial dysfunction and systemic inflammation, while Lp(a) represents an independent driver of atherosclerosis, highlighting the need for targeted and potentially adjunctive therapeutic strategies (Fahed and Jang, 2021; Kittleson, 2025) (Figure 1).

## 7 Pathogenesis of IHD: decoding the molecular and cellular cascade

### 7.1 Endothelial dysfunction as the initiating event

**FIGURE 2:**
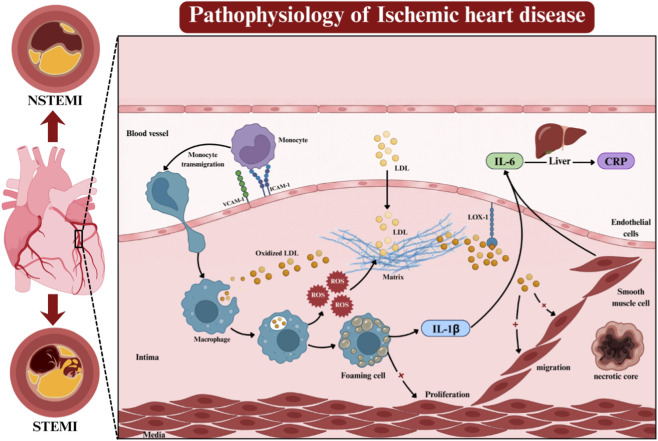
Pathophysiological mechanisms of ischemic heart disease. This figure summarizes the key processes driving ischemic heart disease, beginning with endothelial dysfunction, LDL-c oxidation, and monocyte recruitment, leading to foam cell formation, smooth muscle cell proliferation, necrotic core development, and plaque progression mediated by inflammatory cytokines such as IL-1β and IL-6, with systemic C-reactive protein (CRP) amplification. Prolonged ischemia results in irreversible myocardial injury: ST-elevation myocardial infarction (STEMI) causes transmural necrosis due to complete coronary occlusion, whereas Non-ST-elevation myocardial infarction (NSTEMI) produces subendocardial damage from partial obstruction. Although reperfusion restores blood flow, it may exacerbate injury through oxidative stress, calcium overload, mitochondrial dysfunction, apoptosis, necrosis, and ferroptosis, an iron-dependent lipid peroxidation–driven form of cell death.

Endothelial dysfunction represents an early and critical event in IHD, driven by reduced nitric oxide (NO) bioavailability and increased oxidative stress (Cheng et al., 2023) (Figure 2). Excess reactive oxygen species (ROS), promoted by conditions such as diabetes and dyslipidemia, impair endothelial integrity and activate pro-inflammatory signaling, including upregulation of adhesion molecules vascular cell adhesion molecule-1 (VCAM-1) and intercellular adhesion molecule 1 (ICAM-1), facilitating leukocyte recruitment (Kibel et al., 2020; Juricic et al., 2025). These oxidative and inflammatory processes constitute key therapeutic targets for antioxidant and endothelial-protective phytochemicals.

### 7.2 Atherosclerosis and plaque evolution

Atherosclerosis is initiated by the retention and oxidation of LDL-c within the vascular intima, leading to foam cell formation and early plaque development (Kibel et al., 2020). Progressive lipid accumulation and vascular remodeling contribute to plaque maturation and reduced arterial compliance (Zhang and Dhalla, 2024; Juricic et al., 2025; Buja and Concistrè, 2023). Modulation of lipid oxidation and foam cell formation represents a central mechanism targeted by natural compounds with anti-atherogenic properties.

### 7.3 Inflammation as a central driver

Chronic vascular inflammation plays a pivotal role in plaque progression through cytokine-mediated signaling pathways, including interleukin-6 (IL-6), tumor necrosis factor-alpha (TNF-α), and interleukin 1-beta (IL-1β) (Zhang and Dhalla, 2024). Activation of the NLR family pyrin domain containing 3 (NLRP3) inflammasome further links metabolic dysregulation to vascular injury, while elevated C-reactive protein reflects ongoing inflammatory activity and plaque instability (Zhang and Dhalla, 2024; Severino et al., 2020), these inflammatory pathways are key targets for phytochemicals with anti-inflammatory activity.

### 7.4 Plaque instability and thrombotic complications

The transition from stable disease to acute coronary events is governed by plaque instability and thrombus formation. Disruption of the fibrous cap or endothelial erosion exposes thrombogenic components, triggering platelet activation and occlusive thrombus formation (Nayfeh et al., 2023; Bastiany et al., 2022), where modulation of platelet aggregation and vascular inflammation represents an important therapeutic axis for natural compounds.

### 7.5 Coronary microvascular dysfunction

Coronary microvascular dysfunction contributes to ischemia through impaired vasodilation and structural remodeling, leading to reduced coronary flow reserve (CFR) despite the absence of major arterial obstruction (Cheng et al., 2023; Severino et al., 2020). This condition reflects dysregulation of vascular tone and oxidative stress, highlighting additional targets for phytochemicals that improve endothelial and microvascular function.

### 7.6 Myocardial ischemia and cellular injury

Coronary microvascular dysfunction contributes to ischemia through impaired vasodilation and structural remodeling, leading to reduced CFR despite the absence of major arterial obstruction (Cheng et al., 2023; Severino et al., 2020). This condition reflects dysregulation of vascular tone and oxidative stress, highlighting additional targets for phytochemicals that improve endothelial and microvascular function.

## 8 Key therapeutic nodes in IHD

Although IHD appears complex, its pathogenesis involves interconnected biological processes. Therapeutically, this complexity simplifies to key nodes like oxidative stress, inflammation, endothelial dysfunction, lipid dysregulation, thrombosis, and mitochondrial impairment, which drive vascular and myocardial injury (Table 1). Targeting these nodes offers a more cohesive strategy than focusing on single downstream events. This approach deepens understanding of disease and supports the evaluation of multi-target agents, especially natural bioactive compounds with pleiotropic effects.

**TABLE 1:** Key therapeutic nodes in IHD and their biological roles.

Therapeutic node	Pathophysiological role	Key mediators	Clinical relevance	References
Oxidative stress	Drives endothelial dysfunction, promotes LDL-c oxidation, and amplifies vascular injury	ROS, NOX, Nrf2	Upstream driver of multiple pathological processes	Cheng et al. (2023), Kibel et al. (2020)
Inflammation	Promotes plaque progression, immune activation, and plaque instability	NF-κB, IL-6, TNF-α, NLRP3	Central amplifier of disease progression	Zhang and Dhalla (2024), Severino et al. (2020)
Endothelial dysfunction	Impairs vasodilation, enhances leukocyte adhesion, and disrupts vascular homeostasis	eNOS, NO, VCAM-1, ICAM-1	Early and potentially reversible event	Cheng et al. (2023), Juricic et al. (2025)
Lipid dysregulation	Leads to lipid accumulation, foam cell formation, and plaque development	LDL-c, oxLDL-c, PCSK9	Core mechanism in atherogenesis	Kibel et al. (2020), Buja and Concistrè (2023)
Thrombosis	Causes acute coronary occlusion following plaque rupture or erosion	Platelets, thrombin, TXA2	Immediate trigger of acute events	Fahed and Jang (2021), Kittleson (2025)
Mitochondrial dysfunction	Drives cardiomyocyte death, oxidative injury, and ischemia–reperfusion damage	ROS, Bax/Bcl-2, caspases	Key determinant of myocardial injury	Heusch (2024), Kibel et al. (2020)

## 9 From symptoms to certainty: diagnosing IHD

The diagnosis of IHD primarily distinguishes between acute coronary syndromes (ACS) and chronic coronary syndromes (CCS) based on clinical presentation and biomarker assessment (Kittleson, 2025; Jensen et al., 2020). In ACS, electrocardiography and high-sensitivity cardiac troponin (hs-cTn) are central for detecting myocardial injury, reflecting underlying ischemia-induced cellular damage and necrosis—processes closely linked to oxidative stress and inflammation (Jensen et al., 2020). Emerging biomarkers, such as circulating microRNAs (miR-1, miR-133a, miR-208b, miR-499), further highlight early molecular changes associated with myocardial injury and may provide additional targets for therapeutic modulation (Zalzal et al., 2025; Virani et al., 2023; Dettling et al., 2025).

In CCS, diagnostic approaches focus on identifying functional ischemia and coronary flow impairment through imaging and physiological assessment (Yoshida et al., 2024; Pellikka et al., 2020; Maffeis et al., 2023; Nayfeh et al., 2023). These methods reflect underlying pathophysiological mechanisms, including endothelial dysfunction and microvascular impairment, which are increasingly recognized as targets for both conventional therapies and phytochemical-based interventions (Jensen et al., 2020; Bastiany et al., 2022).

## 10 Current treatment options for IHD

Management of IHD targets key pathological processes, including oxidative stress, inflammation, endothelial dysfunction, lipid accumulation, and thrombosis mechanisms that overlap with those modulated by natural products and phytochemicals (Attiq et al., 2024; Virani et al., 2023). Current therapy integrates pharmacological and interventional approaches to reduce ischemic injury and prevent major adverse cardiovascular events (MACE) (Vrints et al., 2024).

### 10.1 Pharmacological therapy

Pharmacological treatment remains central, acting on multiple mechanistic pathways relevant to disease progression. Antiplatelet and anticoagulant agents reduce thrombus formation by inhibiting platelet activation and coagulation cascades (Sibbing et al., 2023; Navar et al., 2022). Anti-anginal drugs improve myocardial oxygen balance and metabolic efficiency, partly by modulating mitochondrial function and vascular tone (Montelaro et al., 2024; Patel et al., 2025; Johri et al., 2023; Morrisette and Dunn, 2023).

Lipid-lowering therapies, particularly statins, exert both cholesterol-lowering and pleiotropic effects, including suppression of oxidative stress and vascular inflammation via inhibition of NADPH oxidase (NOX) and related signaling pathways (O’Sullivan et al., 2022; Shorbaji et al., 2025). Emerging agents such as proprotein convertase subtilisin/kexin type 9 (PCSK9) inhibitors further enhance plaque stabilization and reduce inflammatory activity (Delialis et al., 2023).

Renin–angiotensin–aldosterone system (RAAS) modulators improve endothelial function and reduce oxidative stress and vascular remodeling (Nádasy et al., 2024; Koumallos et al., 2023; Molaei et al., 2023; Shah et al., 2024). In parallel, glucose-lowering agents such as sodium-glucose transport protein 2 (SGLT2) inhibitors and glucagon-like peptide-1 (GLP-1) receptor agonists provide cardioprotective effects beyond glycemic control by attenuating inflammation, oxidative stress, and metabolic dysfunction (Ostrominski and Vaduganathan, 2024; Park et al., 2024; Nicholls et al., 2025; Nauck and Tirzepatide, 2022).

**FIGURE 3:**
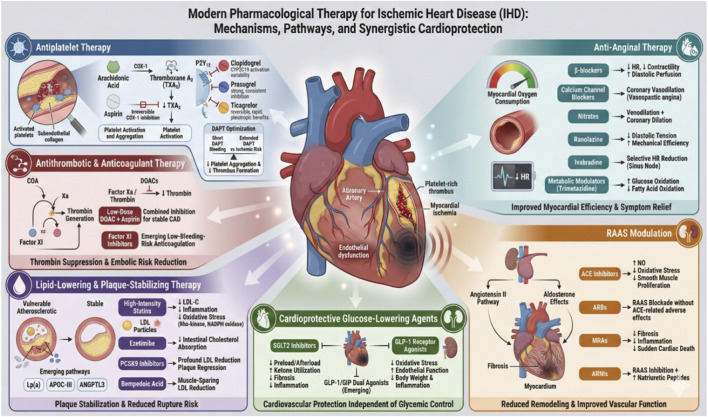
Pharmacological therapy in the management of IHD. This figure summarizes pharmacologic strategies for IHD, highlighting complementary mechanisms that reduce ischemia, thrombosis, inflammation, and adverse remodeling. In addition to antiplatelet, anticoagulant, lipid-lowering, anti-anginal, and RAAS-modulating therapies, cardioprotective glucose-lowering agents have emerged as a major advance in integrated cardiometabolic care. SGLT2 inhibitors reduce preload and afterload, improve myocardial energetics, and attenuate fibrosis and inflammation, while GLP-1 receptor agonists exert anti-atherosclerotic and endothelial-protective effects with favorable impacts on weight and systemic inflammation. Together, these therapies provide cardiovascular protection beyond glycemic control and contribute to improved myocardial efficiency, plaque stabilization, and long-term risk reduction in IHD.

Collectively, these therapies converge on shared mechanistic targets oxidative stress, inflammation, endothelial dysfunction, and metabolic imbalance highlighting parallels with phytochemical-based interventions (Figure 3).

### 10.2 Revascularization strategies

Revascularization approaches, including PCI and CABG, restore myocardial perfusion in patients with advanced disease or persistent ischemia (Nogueira-Garcia et al., 2024; Galli et al., 2023; Yasmin et al., 2024). While effective in improving blood flow, these strategies do not directly address the underlying molecular drivers of IHD, reinforcing the need for complementary therapies targeting inflammation, oxidative stress, and endothelial dysfunction.

## 11 Future directions for the management of IHD

**FIGURE 4:**
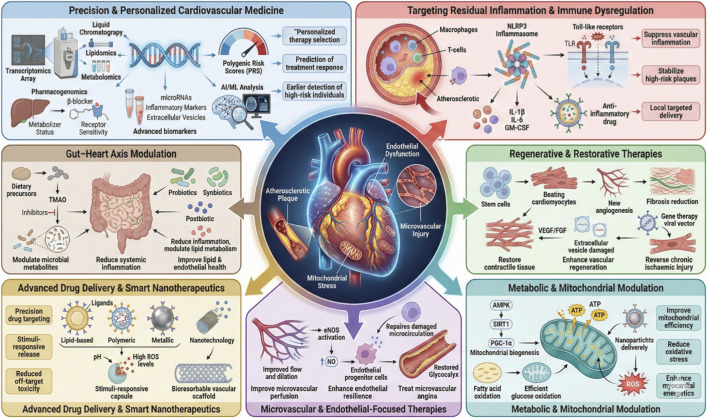
Future directions for the management of IHD. Future IHD therapy is shifting toward precision-based, mechanism-driven strategies that extend beyond conventional risk factor control. Emerging approaches incorporate personalized medicine using multi-omics profiling, pharmacogenomics, biomarkers, and artificial intelligence to refine risk stratification and treatment selection. Targeted immunomodulatory therapies aim to suppress residual vascular inflammation, while regenerative, metabolic, and mitochondrial interventions seek to restore myocardial integrity and energetic efficiency. Microvascular- and endothelial-focused therapies, advanced nanotherapeutics, and gut–heart axis modulation further expand the therapeutic landscape, highlighting a transition toward integrated and restorative cardiovascular care.

Despite major progress in diagnostics and therapy, IHD remains a dominant global health challenge. Many patients continue to develop recurrent angina, advancing atherosclerosis, microvascular injury, and progressive structural dysfunction even with optimized treatment (Figure 4). These gaps underscore the demand for next-generation, mechanism-driven approaches that can modify disease biology across genetic, molecular, metabolic, and microenvironmental domains (Zhao et al., 2024; Wang H. et al., 2025). These emerging strategies also provide a framework for integrating natural compounds and phytochemicals as adjunct or complementary therapies targeting shared molecular pathways.

### 11.1 Precision and personalized cardiovascular medicine

Rapid advances in multi-omics platforms, including genomics, transcriptomics, proteomics, lipidomics, and metabolomics, are reshaping the approach to individualized cardiovascular care. Rather than relying on population-based risk algorithms, precision medicine seeks to tailor therapy based on each patient’s unique molecular profile, biological behavior, and predicted treatment response (Mitsis et al., 2025; Wang R-S. et al., 2023). Additionally, natural products could be integrated into precision medicine frameworks by using pharmacogenomic markers (such as CYP450 polymorphisms) to optimize their metabolism, efficacy, and safety profiles (Rehan and Farooq, 2024).

- Polygenic risk scores (PRS) are expected to play a central role in identifying individuals at heightened genetic susceptibility for premature atherosclerosis, enabling earlier and more aggressive preventive interventions (Fahed and Natarajan, 2023; O’Sullivan et al., 2022).
- Pharmacogenomic testing is anticipated to refine drug selection and dosing for agents such as antiplatelet medications, beta-blockers, and statins by predicting interindividual variability in drug metabolism and receptor sensitivity (Shorbaji et al., 2025).
- Advanced biomarker panels, including inflammatory mediators, endothelial injury markers, microRNAs, and circulating extracellular vesicles, may offer superior accuracy for stratifying patients according to residual risk and guiding therapy intensification (Bernáth-Nagy et al., 2024; Bokhari et al., 2025).

In parallel, artificial intelligence and machine learning are emerging as transformative tools capable of integrating complex clinical, biochemical, and imaging datasets. These technologies are expected to elevate diagnostic precision, detect subclinical disease, forecast adverse events, and facilitate real-time, personalized clinical decision-making (Khalifa and Albadawy, 2024; Simon et al., 2025; Elhaddad and Hamam, 2024).

### 11.2 Targeting residual inflammation and immune dysregulation

Persistent vascular inflammation represents a major driver of plaque progression, instability, and thrombosis, even among patients achieving guideline-recommended LDL-c targets. As a result, next-generation therapies are increasingly centered on immune modulation and targeted inflammation reduction (Zubirán et al., 2024). These pathways overlap with mechanisms targeted by several phytochemicals, including polyphenols and flavonoids, which exhibit immunomodulatory and anti-inflammatory effects.

- Innate immune regulators, including the NLRP3 inflammasome, toll-like receptors (TLRs), and interferon pathways, have become priority targets for reducing chronic vascular inflammation and stabilizing high-risk plaques (Kong et al., 2022)
- Selective cytokine-targeted therapies, such as inhibitors of IL-1β, IL-6, and granulocyte–macrophage colony-stimulating factor (GM-CSF), are under investigation to suppress specific inflammatory pathways with fewer systemic adverse effects than broad immunosuppressive agents (Narazaki and Kishimoto, 2022; Wang Y. et al., 2022).
- Nanoparticle-enabled drug delivery systems promise to localize anti-inflammatory treatments directly to lipid-rich or inflamed plaques, minimizing systemic exposure while maximizing intraplaque therapeutic concentration. By directly addressing the inflammatory component of atherosclerosis, these therapies have the potential to reshape long-term cardiovascular risk reduction (Karami et al., 2023; Chen et al., 2022).

### 11.3 Regenerative and restorative therapies

Regenerative cardiology is rapidly expanding as researchers seek to restore, rather than merely protect, injured myocardial tissue. These innovative strategies aim to promote structural healing, enhance vascular regeneration, and recover contractile function (Karami et al., 2023; Chen et al., 2022; Karim and Tompkins, 2025).

- Stem cell–derived therapies, including mesenchymal stem cells, adipose-derived stromal cells, cardiac progenitor cells, and induced pluripotent stem cells, are being investigated for their ability to differentiate into cardiomyocytes, stimulate angiogenesis, and reduce fibrosis (Yang J. et al., 2025).
- Extracellular vesicle and exosome-based therapies are emerging as powerful acellular alternatives that deliver microRNAs, growth factors, and cytoprotective signals capable of enhancing myocardial survival and microvascular repair (Ostrominski and Vaduganathan, 2024; Presume et al., 2024; Carlin et al., 2024).
- Cardiac gene therapy, using vectors encoding pro-angiogenic factors (e.g., vascular endothelial growth factor (VEGF), fibroblast growth factor (FGF)), anti-apoptotic mediators, or mitochondrial regulators, offers promising potential to improve perfusion in chronically ischemic myocardium and counteract cellular injury. Collectively, these restorative strategies represent a paradigm shift toward functional and structural myocardial rejuvenation (Sun et al., 2025; Lee et al., 2024).

### 11.4 Metabolic and mitochondrial modulation

Growing evidence indicates that metabolic inflexibility and mitochondrial dysfunction are critical contributors to ischemic injury, cardiomyocyte death, and post-infarction remodeling. Future therapeutic approaches increasingly aim to repair mitochondrial integrity and optimize myocardial energetics (Shi et al., 2025). Notably, natural compounds such as resveratrol, curcumin, and quercetin have been shown to modulate AMP-activated protein kinase/sirtuin-1 (AMPK/SIRT1) pathways and mitochondrial function, aligning with these emerging therapeutic strategies (Hamidu et al., 2026).

- Metabolic modulators are being developed to promote a shift toward more oxygen-efficient glucose utilization pathways, thereby reducing myocardial oxygen consumption during stress (Fischer et al., 2025).
- Activation of mitochondrial biogenesis via AMPK, SIRT1, and peroxisome proliferator–activated receptor-gamma coactivator 1-alpha (PGC-1α) signaling pathways holds potential for enhancing ATP production, improving oxidative phosphorylation, and strengthening cardiomyocyte resilience (Zong et al., 2024).
- Mitochondria-targeted antioxidants, including novel small molecules and nanoparticle-encapsulated compounds, are expected to neutralize ROS within the mitochondrial matrix more effectively than systemic antioxidants. These metabolic interventions aim to preserve myocardial viability, limit infarct expansion, and enhance recovery following acute or chronic ischemia (Nahar and Sohag, 2025; Li et al., 2025).

### 11.5 Microvascular and endothelial-focused therapies

Microvascular dysfunction and endothelial impairment are increasingly recognized as central drivers of persistent angina, ischemia, and reduced CFR, particularly in patients without obstructive epicardial disease. As a result, emerging research is focusing on strategies that specifically target the microcirculatory environment (Spione et al., 2022; Hwang et al., 2023). This is particularly relevant to phytochemicals known to enhance NO bioavailability and reduce oxidative stress (Muscolo et al., 2024).

- Endothelial NO–enhancing agents, such as NO donors, endothelial NO synthase (eNOS)-stimulating compounds, and cofactors that restore NO bioavailability, hold promise for improving vasodilation and microvascular perfusion (Hwang et al., 2023).
- Endothelial progenitor cell (EPC) therapy aims to repair damaged microvascular networks and regenerate endothelial surfaces disrupted by oxidative and inflammatory stress (Huang et al., 2022).
- Glycocalyx-restoring therapies, including sulfated polysaccharides and antioxidant formulations, may protect this fragile endothelial barrier and improve vascular permeability and shear-stress responsiveness (Ying et al., 2023; Gimblet et al., 2024). These targeted approaches address key pathophysiological mechanisms underlying microvascular angina and persistent ischemia.

### 11.6 Advanced drug delivery and smart nanotherapeutics

Breakthroughs in material science and nanotechnology are enabling unprecedented precision in cardiovascular drug delivery. These innovations aim to maximize therapeutic efficacy while minimizing off-target toxicity (Tapeinos et al., 2022; Omidian et al., 2023).

- Lipid-based, polymeric, and metallic nanocarriers are being engineered with specialized surface ligands that recognize plaque-associated biomarkers, allowing selective accumulation within atheromatous lesions (Bartusik-Aebisher et al., 2025). In parallel, stimuli-responsive delivery platforms capable of releasing therapeutic agents in response to localized changes in pH, oxidative stress, or enzymatic activity are poised to refine drug-release timing and enhance treatment specificity (Zhang X. et al., 2024). Additionally, bioresorbable vascular scaffolds and micro-implantable drug reservoirs provide targeted, sustained delivery of therapeutic agents while circumventing the long-term drawbacks associated with permanent stent implantation, collectively shaping a new era of precision cardiovascular therapeutics (Drelich and Goldman, 2022; Magill et al., 2023). These technologies reflect the growing movement toward targeted, minimally invasive, and physiologically adaptive cardiovascular interventions.

### 11.7 Gut–heart axis modulation

Emerging evidence highlights the powerful and bidirectional relationship between gut microbial composition and cardiovascular health. Microbial metabolites, immune signaling, and intestinal barrier function all influence the development and progression of atherosclerosis (Mohsenzadeh et al., 2025). Dietary polyphenols and plant-derived bioactives play a key role in modulating gut microbiota composition and metabolite production (Aravind et al., 2021).

- Targeted modulation of gut microbiota through probiotics, prebiotics, synbiotics, or dietary interventions may shift the microbial balance toward cardioprotective phenotypes (Lei et al., 2023; Yu et al., 2025).
- Enzyme inhibitors targeting microbial pathways responsible for producing pro-atherogenic metabolites such as trimethylamine N-oxide (TMAO) are being investigated as novel therapeutic strategies (Schugar et al., 2022).
- Postbiotic therapies, including microbial-derived metabolites and bioactive compounds, may deliver anti-inflammatory, lipid-modulating, and endothelial-protective benefits independent of live microbial administration. By addressing microbiome-derived contributors to systemic inflammation and lipid dysregulation, gut–heart axis modulation offers a promising and expanding frontier in cardiovascular prevention (Flori et al., 2024; Snelson et al., 2025).

Collectively, these future directions highlight multiple converging pathways—oxidative stress, inflammation, metabolic dysfunction, and endothelial impairment—that are also modulated by natural compounds, reinforcing their potential role as complementary strategies in next-generation IHD management.

## 12 Lifestyle and environmental factors in the development and management of IHD

**FIGURE 5:**
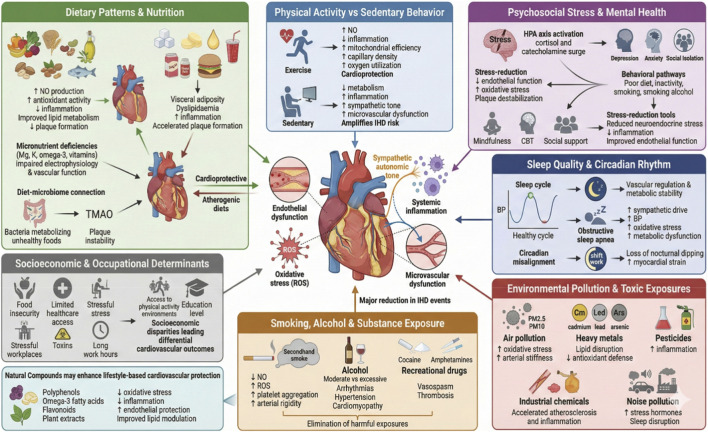
Lifestyle and environmental factors influencing IHD. Lifestyle habits and environmental exposures play central roles in the development and progression of ischemic heart disease by regulating endothelial function, inflammation, oxidative stress, metabolic balance, and myocardial resilience. Dietary quality, physical activity, psychosocial stress, sleep patterns, and substance exposure directly influence atherogenesis and vascular dysfunction, while environmental pollution and socioeconomic factors further modulate cardiovascular risk. Optimization of these factors enhances prevention, supports pharmacological therapy, and provides mechanistic insight into integrative and natural compound–based cardiovascular strategies.

Lifestyle factors, particularly dietary patterns, play a critical role in modulating key pathophysiological mechanisms in IHD, including oxidative stress, inflammation, endothelial dysfunction, and metabolic imbalance (Figure 5). Importantly, many of these effects are mediated through the intake of bioactive compounds such as polyphenols, flavonoids, and other phytochemicals, which form the basis of natural therapeutic strategies. Thus, lifestyle modification not only reduces risk but also enhances the efficacy of both pharmacological and phytochemical-based interventions (Scimeca et al., 2024; Ghodeshwar et al., 2023).

### 12.1 Dietary patterns and phytochemical intake

Diet represents the primary source of cardioprotective phytochemicals. Diets rich in fruits, vegetables, whole grains, legumes, nuts, and unsaturated fats such as the Mediterranean diet—provide abundant polyphenols and flavonoids that enhance NO bioavailability, reduce oxidative stress, and modulate inflammatory signaling pathways (Reyneke et al., 2025; Trinquet et al., 2025; Almevall et al., 2025). These compounds contribute to improved endothelial function, reduced lipid oxidation, and attenuation of atherosclerotic progression.

In contrast, diets high in ultra-processed foods, refined sugars, and saturated fats promote oxidative stress, inflammation, and metabolic dysregulation, accelerating vascular injury (Yang et al., 2024). Emerging evidence also highlights diet–microbiome interactions, where dietary polyphenols and fiber influence gut microbial metabolites such as TMAO, linking nutrition to endothelial dysfunction and plaque instability (Zheng et al., 2024; Wan et al., 2025). Sustained adherence to phytochemical-rich dietary patterns supports vascular homeostasis and enhances cardiometabolic resilience (Llamas-Ramos et al., 2025; Cruijsen et al., 2025).

### 12.2 Lifestyle factors modulating phytochemical efficacy

Additional lifestyle factors influence cardiovascular health through mechanisms overlapping with phytochemical activity. Physical activity enhances endothelial NO production, reduces inflammatory signaling, and improves mitochondrial function, thereby complementing antioxidant and anti-inflammatory effects of natural compounds (Mølmen et al., 2025; Muley et al., 2025; Franssen et al., 2025).

Conversely, environmental and behavioral stressors including smoking and air pollution promote oxidative stress, endothelial injury, and inflammation, counteracting the beneficial effects of both pharmacological and phytochemical interventions (Münzel et al., 2025; Pan et al., 2024; Rahman et al., 2025). These factors highlight the importance of minimizing pro-oxidant exposures to optimize therapeutic outcomes.

## 13 Relevance of natural bioactive compounds within integrative strategies for the management of IHD

**FIGURE 6:**
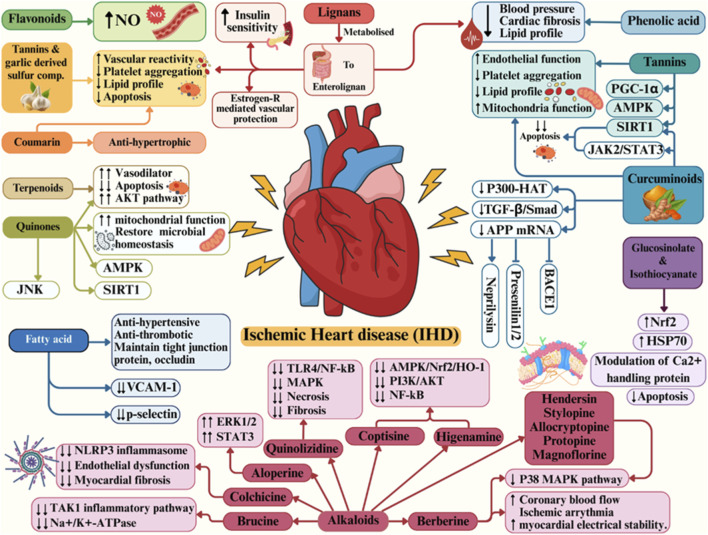
Cardioprotective mechanisms of major phytochemical classes in ischemic heart disease (IHD). This figure summarizes the multitarget mechanisms by which major phytochemical classes confer cardioprotection in IHD. Polyphenols—including flavonoids, phenolic acids, tannins, stilbenes, and lignans—enhance endothelial nitric oxide (NO) production, improve vascular reactivity, reduce platelet aggregation, modulate lipid profiles, and attenuate oxidative stress and inflammation through pathways such as AMPK, SIRT1, PGC-1α, JAK2/STAT3, and Nrf2. Curcuminoids regulate epigenetic and pro-fibrotic signaling (p300-HAT, TGF-β/Smad), suppress apoptosis, and improve post-ischemic remodeling. Quinones, saponins, alkaloids, and terpenoids exert antioxidant, anti-inflammatory, mitochondrial-protective, anti-apoptotic, and ion-channel–modulating effects, targeting pathways including PI3K/Akt, MAPK, NF-κB, and NLRP3 inflammasome signaling. Organosulfur compounds and isothiocyanates activate cytoprotective Nrf2-dependent responses, improve calcium handling, and mitigate ischemia–reperfusion injury, while omega-3 and omega-9 fatty acids enhance endothelial integrity, reduce thrombosis, suppress adhesion molecules, and improve lipid metabolism. Collectively, these natural compounds converge on key processes—oxidative stress, inflammation, endothelial dysfunction, apoptosis, fibrosis, and metabolic imbalance—highlighting their integrative therapeutic potential in the prevention and management of IHD.

Natural bioactive compounds represent a mechanistically relevant extension of integrative strategies for IHD, as their biological activities closely mirror pathways influenced by lifestyle and environmental modulation. Many phytochemicals exhibit antioxidant, anti-inflammatory, endothelial-protective, lipid-modulating, and metabolic-regulating properties, targeting core pathophysiological processes underlying IHD. Within an integrative framework, these compounds may complement conventional therapies and lifestyle interventions, contributing to improved vascular function, reduced inflammation, and enhanced cardiometabolic balance. This convergence supports their role as adjunctive strategies for prevention and disease management (Kaçar et al., 2025; Alum, 2025) (Figure 6).

### 13.1 Major phytochemical classes and cardiovascular relevance

#### 13.1.1 Polyphenols

Flavonoids emerged as promising multi-target cardioprotective agents. Several flavonoids demonstrated an effective role in the management of IHD include flavonols; quercetin (*Allium species*, *Vitis species*, and *Malus domestica*) and kaempferol (*Camellia sinensis*, *Spinacia oleracea*, *Lactuca sativa*, *Brassica oleracea*, and other leafy vegetables), flavanols; catechin and epicatechin (*C. sinensis*), flavones; luteolin and apigenin (*Apium graveolens*, P*etroselinum crispum*, *Daucus carota*, *Capsicum annuum*, *Thymus vulgaris*, *Mentha piperita*, and *Matricaria chamomilla*), flavanones; naringenin and hesperetin (*Citrus species*), isoflavones; genistein, and daidzein (*Glycine max*), and anthocyanins (*Fragaria ananassa*, *Vaccinium species*, *Rubus idaeus*, and other berries). These compounds were reported to have the ability to enhance endothelial NO production, improve vascular relaxation, and thus reduce myocardial injury, enhance coronary perfusion, and mitigate ischemia–reperfusion damage via their antioxidant and anti-inflammatory effects (Liu et al., 2024; Ciumărnean et al., 2020; Mansour et al., 2024; Fardoun et al., 2020; Fardoun et al., 2019). Epidemiological evidence indicates that high intake of flavonoids is linked to a reduced risk of myocardial infarction and IHD-related mortality, with reported relative risk reductions (RR) for quercetin and catechin of 0.79 and 0.76, respectively (Gross, 2004).

##### 13.1.1.1 Phenolic acids

Phenolic acids are promising natural compounds for the prevention and management of IHD through diverse cardiovascular protective mechanisms. Hydroxybenzoic acids; gallic acid and protocatechuic acid (*V. species*, *F. ananassa*, *V. species*, *R. idaeus*, *G. max*, *Arachis hypogaea*, *Juglans regia*, *Carya illinoinensis*, *Sesamum indicum*, *Allium species*, *Quercus alba*, *C. annuum*, *C. sinensis*, *Salvia rosmarinus*, and *Hibiscus sabdariffa*) and hydroxycinnamic acids; rosmarinic acid (*Rosmarinus officinalis*, *Salvia officinalis*, *T. vulgaris*, *M. piperita*, and *Ocimum basilicum*), chlorogenic acid and caffeic acid (*Coffea arabica*, *M. domestica*, *Pyrus communis*, *Prunus species*, *V. species*, *Cynara scolymus*, *Solanum species*, and *M. chamomilla*), and ferulic acid (*Triticum aestivum*, *Avena sativa*, *Zea mays*, *Oryza sativa*, *Linum usitatissimum*, *S. indicum*, *S. oleracea*, *D. carota*, *C. species*, and *M. domestica*) demonstrated cardioprotective effects via attenuating cardiac fibrosis and hypertrophy, reducing myocardial infarction, alleviating myocardial injury, lowering blood pressure, and improving lipid profiles, through their antioxidant and anti-inflammatory activities (Ali et al., 2020; Cheng et al., 2017).

##### 13.1.1.2 Tannins

Tannins act through multi-target mechanisms to manage and prevent IHD. Condensed tannins; procyanidins (A and B-type) (*V. species*, *Theobroma cacao*, *C. sinensis*, *Vaccinium macrocarpon*, and *Pinus maritima*), hydrolysable gallotannins; epigallocatechin gallate (EGCG) (*C. sinensis*), penta-O-galloyl-β-D-glucopyranose (PGG) (*Rhus coriaria*), tannic acid (Quercus *species* and *R. coriaria*), and hydrolysable ellagitannins; punicalagin (*Punica granatum*) exert cardiovascular benefits through improving vascular reactivity, reducing platelet aggregation, improving lipid profiles and coronary perfusion pressure, attenuating myocardial injury, and protection against ischemia–reperfusion injury, via their antioxidant, anti-inflammatory, and apoptosis-related signaling pathways inhibition activities (Koleckar et al., 2008; Beretta et al., 2009; Hu et al., 2015).

##### 13.1.1.3 Stilbenes

Resveratrol (3,5,4′-trihydroxy-trans-stilbene) is a naturally occurring stilbene found in *V. species*, *V. species*, *A. hypogaea*, *T. cacao*, and *Polygonum cuspidatum*, and represents a promising adjunctive therapeutic and preventive agent for IHD. Resveratrol demonstrated multitarget cardioprotective mechanisms, including improvement of endothelial function and lipid profile, mitigation of atherosclerotic progression, and inhibition of platelet aggregation. It activates primary molecular regulators; SIRT1, AMPK, and PGC-1α, promoting mitochondrial function and limiting ischemia–reperfusion injury, through its antioxidant, anti-inflammatory, and vascular protective mechanisms (Dyck et al., 2019).

##### 13.1.1.4 Lignans

Lignans are plant-derived phenolic compounds that demonstrated potential involvement in the prevention and treatment of IHD, particularly CHD (Raj et al., 2021). Dibenzylbutane lignans; secoisolariciresinol and lariciresinol, dibenzylbutyrolactone lignans; metaraminol, furofuran lignans; pinoresinol, syringaresinol, matairesinol, and sesamin were found to be rich in *Linum usitatissimum*, *S. indicum*, *Secale cereale*, *T. aestivum*, *A. sativa*, *G. max*, *Cicer arietinum*, *Lens culinaris*, *F. ananassa*, *V. species*, *Brassica species*, *D. carota*, *Olea europaea*, *V. species*, *C. sinensis*, and *C. arabica*. Following ingestion, these lignans are metabolized via gut microbiota into the bioactive enterolignans enterodiol and enterolactone. Enterolignans exert multifaceted cardioprotective effects relevant to IHD, including inhibition of platelet aggregation, improvement of lipid profile, improvement of blood pressure, attenuation of vascular inflammation, inhibition of atherogenic processes, and estrogen receptor–mediated vascular protection, through antioxidant, anti-inflammatory, endothelial, antithrombotic, and phytoestrogenic mechanisms. Enterolignans have also been associated with improvements in endothelial function, insulin sensitivity, and vascular stiffness, which are relevant to IHD. Clinically, the hazard ratios for CHD for total lignans, matairesinol, secoisolariciresinol, pinoresinol, and lariciresinol were 0.85, 0.76, 0.87, 0.89, and 0.89, respectively, indicating a strong inverse association. Also, enterolignans showed synergistic interactions with dietary fiber intake in strengthening CHD risk reduction (Laveriano-Santos et al., 2025; Hu et al., 2021).

#### 13.1.2 Coumarins

Major coumarins implicated in IHD include simple coumarins; coumarin (*Cinnamomum cassia* and *Melilotus officinalis*), umbelliferone (*Ammi majus*, *Coriandrum sativum*, *Ferula species*, *C. species*, *Angelica species*, *Aegle marmelos*, and *Pilosella officinarum*), esculetin (*Aesculus hippocastanum*), and scopoletin (*Morinda citrifolia* and *F. species*). Also, furanocoumarins; psoralen (*A. majus* and *A. species*), bergapten (*A. species*, *F. species*, and *C. species*), pyranocoumarins; seselin (*A. species* and *F. species*), decursin (*A. species*), and praeruptorins (*Peucedanum species*) are included. These coumarins were reported to exert cardioprotective effects relevant to IHD including, anticoagulant, antithrombotic, antihyperlipidemic, antihypertrophic, and vasodilatory activities, protecting against ischemia–reperfusion–induced myocardial injury, throughout their antioxidant and anti-inflammatory properties (Najmanova et al., 2015; Jagadeesh et al., 2016).

#### 13.1.3 Cannabinoids

The role of cannabinoids in IHD has been investigated highlighting their cardioprotective mechanisms *via* CB2 receptor activation, modulation of inflammation, and reduction of oxidative stress with modulation of SIRT-1/PGC-1α-related mitochondrial biogenesis and NF-κB-dependent inflammatory signaling, in addition to modulation of adipocyte biology, regional fat distribution, and atherosclerosis, as well as precipitation of hemodynamic stressors relevant in the setting of myocardial infarction (Aksoy et al., 2022; Seif El Dahan et al., 2022).

#### 13.1.4 Curcuminoids

Curcuminoids are bioactive compounds, dominated by curcumin, derived from the rhizomes of *Curcuma longa* and have been widely studied for their cardioprotective potential in IHD. Curcumin protects against myocardial ischemia–reperfusion injury through improving endothelial function and lipid profiles, exerting pleiotropic protective effects relevant to IHD, and limiting cardiomyocyte apoptosis via activating SIRT1 and janus kinase 2/signal transducer and activator of transcription 3 signaling pathway (JAK2/STAT3) signaling, downregulating the messenger RNA (mRNA) expression of amyloid precursor protein (APP) and its amyloidogenic processing enzymes β-secretase-1 (BACE1), presenilin-1, and presenilin-2, and increasing the expression of neprilysin (Cox et al., 2022; Sayevand et al., 2022). It also improves post-ischemic injury ventricular remodeling via suppressing p300 histone acetyltransferase (p300-HAT) activity and transforming growth factor–beta/smad signaling pathway (TGF-β/Smad) signaling (Yang et al., 2024). These cardioprotective effects are mediated through its antioxidant, anti-inflammatory, mitochondrial-protective, anti-apoptotic, anti-amyloidogenic, and gene-regulatory mechanisms (Cox et al., 2022; Yang et al., 2024).

#### 13.1.5 Quinones

Several quinones showed cardioprotective relevance in IHD including anthraquinones; emodin, emodin glucoside, aloe-emodin, aloe-emodin glucoside, chrysophanol, chrysophanol glucoside, physcion glucoside (*Aloe species*, *Rheum species*, and *Polygonum species*), rhein, rhein glucoside, danthron (*R. species* and *Polygonum species*), purpurin (*Rubia tinctorum*), and hypericin (*Hypericum perforatum*), naphthoquinones; plumbagin (*Plumbago species*) and shikonin (*Lithospermum erythrorhizon*), and benzoquinones; embelin (*Embelia ribes*) (Akanda et al., 2025; Li Q. et al., 2020). The anthraquinone glycosides reported are not directly absorbed in glycosidic form, but are metabolized by intestinal flora into their corresponding anthraquinone aglycones (Li Q. et al., 2020). Quinones modulate mitochondrial function, regulate AMPK, phosphoinositide 3-kinase/protein kinas B (PI3K/Akt), c-Jun N-terminal kinase (JNK), and SIRT1 pathways, improve endothelial and coronary function, inhibit cardiomyocyte apoptosis, and thus protect against myocardial ischemia and ischemia–reperfusion injury (Akanda et al., 2025). These effects are mediated via their antioxidant, anti-inflammatory, anti-apoptotic, and restoration of microbial homeostasis mechanisms (Akanda et al., 2025; Li Q. et al., 2020).

#### 13.1.6 Saponins

Saponins demonstrated significant multitarget cardioprotective and therapeutic potentials in IHD, including triterpenoid saponins; notoginsenoside R1, ginsenosides Rb1, Rg1, Rd, and Rg3 (*Panax notoginseng* and *Panax ginseng*), astragaloside IV (*Astragalus propinquuos*), and chikusetsusaponin V, IV, IVa, and pseudoginsenoside RT1 (*Panacis majoris*). These saponins exert their protective effects against myocardial infarction, myocardial ischemia, ischemia–reperfusion injury, and heart failure through antioxidant, anti-inflammatory, anti-apoptotic, autophagy-regulating, hemorheological, antiplatelet, and metabolic mechanisms (Ma et al., 2022; Li M. et al., 2020).

#### 13.1.7 Alkaloids

Alkaloids are a diverse class of naturally occurring nitrogen-containing compounds widely recognized for their multitarget therapeutic potential in IHD, including myocardial infarction, ischemia-induced heart failure, and post-ischemic cardiac remodeling via their anti-inflammatory, antioxidant, ion-channel–modulating, and anti-remodeling mechanisms (Prajapati and Shah, 2025). Isoquinoline alkaloids, such as berberine (*Berberis vulgaris* and *Corydalis hendersonii*), improve myocardial ischemia by enhancing coronary blood flow, reducing ischemic arrhythmias, and improving myocardial electrical stability, mediated by its anti-inflammatory, anti-oxidant, and anti-apoptotic properties, as evidenced by several ischemia-reperfusion animal models (Wang et al., 2018; Yang H. et al., 2025; Zhu et al., 2017). Higenamine (*Aconitum species*) that also activates PI3K/Akt pathway and suppresses nuclear factor-kappa B (NF-κB) signaling, magnoflorine, protopine, allocryptopine, stylopine, bicuculline, and hendersine B (*C. hendersonii*) which reduce inflammatory infiltration, myocardial fibrosis, and structural damage via the inhibition of the p38 mitogen-activated protein kinase (MAPK) signaling pathway (Prajapati and Shah, 2025; Wang et al., 2021; Ge et al., 2023). Quinolizidine alkaloids; oxymatrine (*Sophora flavescens*) attenuates myocardial damage by inhibiting inflammatory signaling, reducing oxidative stress, necrosis, and fibrosis, and aloperine (*Sophora alopecuroides*) that activates extracellular signal–regulated kinase 1 and 2 (ERK1/2) and STAT-3 pathways and modulates cardiac repolarization (Prajapati and Shah, 2025) (Hu et al., 2023). Tropolone alkaloids; colchicine (*Colchicum autumnale*) suppresses NLRP3 inflammasome activation, thereby mitigating ischemia-related inflammation, endothelial dysfunction, and myocardial fibrosis (Prajapati and Shah, 2025). Indole alkaloids; brucine (*Strychnos nux-vomica*) reduces ischemic injury via Na^+^/K^+^-ATPase inhibition and tabersonine (*Catharanthus roseus*) that limits post-ischemic cardiac remodeling by targeting transforming growth factor-β-activated kinase 1 (TAK1)-mediated inflammatory signaling (Prajapati and Shah, 2025).

#### 13.1.8 Terpenoids

Terpenoids have been extensively investigated for their cardiovascular pharmacological effects relevant to IHD; monoterpenoids; limonene (*Citrus* spp.), α-pinene and β-pinene (*Eucalyptus* spp. and *Cinnamomum* spp.), borneol (*Cinnamomum* spp. and *Blumea* spp.), carvacrol (*Origanum* spp. and *Croton* spp.), citronellol (*Cymbopogon* spp. and *Eucalyptus* spp.), geraniol (*Cymbopogon* spp. and *Ocimum* spp.), linalool (*Lavandula angustifolia*, *Ocimum* spp. and *Coriandrum* spp.), menthol (*Mentha* spp.), α-terpineol (*Citrus* spp. and *Eucalyptus* spp.), thymol (*Thymus* spp. and *Ocimum* spp.), anethole (*Foeniculum vulgare* and *Illicium verum*), cinnamaldehyde (*Cinnamomum* spp.), eugenol (*Cinnamomum* spp. and *Ocimum* spp.), citral (*Cymbopogon* spp. and *Lippia alba*), citronellal (*Eucalyptus* spp. and *Cymbopogon* spp.), and eucalyptol (*Eucalyptus* spp. and *Alpinia zerumbet*). They exert vasodilator, cardio-modulatory effects relevant to coronary vasoconstriction, impaired myocardial perfusion, and endothelial dysfunction, through calcium channel blockade and endothelial modulation (Cardoso-Teixeira et al., 2020). Triterpenoids; darutigenol (*Siegesbeckia* spp.) has a protective role in case of myocardial infarction and ischemia/reperfusion injury through antioxidant effects, promoting cardiomyocyte survival, reducing apoptosis, and ameliorating remodeling following ischemic injury (Liu K. et al., 2025). Triterpenoids, phytosterols include brassicasterol (*Brassica* spp.), β-sitosterol, campesterol, stigmasterol, and avenasterol, and stanols include sitostanol and campestanol (*G. max*, *Helianthus annuus*, *Z. mays*, *O. europaea*, *A. hypogaea*, *Prunus dulcis*, *S. indicum*, *Linum usitatissimum*, *Cucurbita pepo*, *T. aestivum*, *S. cereale*, and *A. sativa*) reduce serum LDL-c, lowering circulating cholesterol levels (Makhmudova et al., 2021; Poli et al., 2021). Elevated plasma levels of these compounds were found to be associated with an increased risk of IHD through promoting macrophage necrosis, plaque instability, vascular dysfunction, and potential atherogenicity (Makhmudova et al., 2021; Zhao et al., 2023). Tetraterpenoids; carotenoids include lycopene (*Solanum lycopersicum*), β-carotene (*D. carota*, *Ipomoea batatas*, *Brassica* spp., and *S. oleracea*), α-carotene (*D. carota* and *C. pepo*), lutein (*Brassica* spp., and *S. oleracea*), and β-cryptoxanthin (*Citrus* spp. and *Carica papaya*) were found to be inversely associated with the prevalence of IHD and related cardiovascular outcomes, through antioxidant and anti-inflammatory mechanisms (Wang M. et al., 2023).

#### 13.1.9 Organosulfur compounds

##### 13.1.9.1 Garlic-derived sulfur compounds

Garlic-derived sulfur compounds are bioactive natural compounds derived from *Allium sativum,* including alliin, allicin, diallyl sulfide, diallyl disulfide, diallyl trisulfide, ajoene, S-allyl cysteine, and S-allyl mercaptocysteine. These compounds demonstrated a potential role in the prevention and adjunctive management of IHD, especially myocardial ischemia-reperfusion injury, throughout their antioxidant, anti-inflammatory, antihyperlipidemic, anti-thrombotic, vasodilatory, and antiapoptotic mechanisms that mediate myocardial protection and attenuate atherosclerosis (Seki and Hosono, 2025; Wang C. et al., 2025).

#### 13.1.10 Glucosinolate and isothiocyanates

Isothiocyanates were investigated for their cardioprotective effects and in the treatment of IHD, especially myocardial infarction and ischemia-reperfusion injury. Sulforaphane represents a bioactive isothiocyanate, derived from cruciferous vegetables, including *Brassica* spp. and *Nasturtium officinale*, and demonstrated antioxidant, anti-inflammatory, cytoprotective mechanisms, attenuating myocardial infarction injury, and modulation of calcium-handling proteins, improving myocardial contractility and relaxation during ischemia–reperfusion (Zhang et al., 2023; Bonetto et al., 2022). Phenylethyl isothiocyanate is a bioactive isothiocyanate generated through myrosinase-mediated hydrolysis of gluconasturtiin, a glucosinolate found in cruciferous vegetables, including *Brassica* spp., *N. officinale*, and *Raphanus sativus,* that plays a key role in cardiovascular protection relevant to IHD, particularly through modulation of atherosclerosis-related processes via its antioxidant, anti-inflammatory, anti-apoptotic, and anti-atherosclerotic effects (Kamal et al., 2022).

#### 13.1.11 Fatty acids

Omega-3 fatty acids are polyunsaturated fatty acids found in various plants, demonstrated cardioprotective role in IHD. α-Linolenic acid, stearidonic acid, eicosapentaenoic acid, docosapentaenoic acid, and docosahexaenoic acid (*Linum usitatissimum*, *G. max*, *J. regia*, *Salvia hispanica*, *Brassica napus*, *Perilla frutescens*, *Camelina sativa*, *Buglossoides arvensis*, *Echium plantagineum*, and *Ribes nigrum*) exert tissue protective effects following ischemia–reperfusion injury through multitarget mechanisms, including anti-inflammatory; lowers TNF-α levels, antihyperlipidemic; reduces levels of cholesterol, LDL-c, and triglycerides, antihypertensive and anti-thrombotic; improves vascular function, endothelial-protective; maintains the tight-junction protein occludin, and tissue-stabilizing; suppresses VCAM-1 and P-selectin (Bonetti et al., 2021; Prasad et al., 2021). Also, Omega-9 fatty acids, the monounsaturated fatty acids, showed a critical cardioprotective role in the prevention and modulation of IHD, particularly atherosclerosis. Oleic acid (*O. europaea*) confers significant cardioprotective effects against ischemic myocardial injury primarily through antioxidant, anti-inflammatory, antihyperlipidemic, antihypertensive, and anti-atherosclerotic mechanisms, as well as significant promotion of mitochondrial reactivity and cardiac metabolic efficiency (Singh et al., 2020; Lu et al., 2024).

### 13.2 Critical assessment of preclinical evidence and clinical translation gaps

Despite extensive preclinical evidence demonstrating the cardioprotective effects of natural bioactive compounds in IHD, significant barriers limit their clinical translation. Most available data are derived from *in vitro* and small-animal models, which often lack standardized protocols, reproducibility across laboratories, and validation in large-animal systems, thereby limiting their relevance to human disease (Sorkin et al., 2019). Although these studies consistently highlight key mechanisms such as modulation of oxidative stress, inflammation, and endothelial dysfunction, the overall strength and reliability of evidence remain heterogeneous.

A major translational challenge relates to dose relevance and pharmacokinetics (Rao et al., 2019). Many studies employ supraphysiological doses that are not achievable in humans, while compounds such as resveratrol and curcumin suffer from poor aqueous solubility, extensive first-pass metabolism, and low systemic bioavailability, resulting in subtherapeutic plasma concentrations (Siripruekpong et al., 2024). Efforts to address these limitations include the use of human-equivalent dosing (HED) calculations and advanced delivery systems (Saran et al., 2026); however, such strategies remain largely confined to early-stage research and lack standardized translational frameworks.

Another key limitation is the scarcity of robust clinical evidence (Paller et al., 2016). While certain compounds such as berberine, flavonoids, and phytosterols are supported by human or epidemiological data (Usama et al., 2025), many others, including sulforaphane, curcumin, and saponins, remain restricted to preclinical evaluation (Joshi et al., 2025; Panknin et al., 2023). Additionally, the predominance of rodent models and the lack of direct comparisons between conventional and optimized formulations further complicate assessment of true therapeutic efficacy.

To address these gaps, a structured evaluation of lead compounds (Table 2) integrates evidence strength, bioavailability constraints, HED, and translational readiness. Compounds with favorable pharmacokinetic profiles and existing human data (e.g., berberine, catechins, phytosterols) represent the most suitable candidates for early-phase clinical trials, whereas others require further optimization.

**TABLE 2:** Critical evaluation of lead phytochemicals for clinical advancement in IHD.

Compound	Evidence level	Human data availability	Bioavailability	Dose feasibility	Key limitation	Translational readiness	Ref
Berberine	Preclinical + Clinical	Yes	Moderate	Feasible (500–1,500 mg/day)	Variable PK, GI effects	High (Phase II candidate)	Mana et al. (2023)
Phytosterols	Clinical	Yes	Good	Feasible (1,500–3,000 mg/day)	Modest efficacy magnitude	High (clinically established adjunct)	Salehi B. et al. (2021)
Catechins (EGCG)	Preclinical + Clinical	Yes	Moderate	Feasible (250–800 mg/day)	Dose variability	Moderate–High	Ferrari and Naponelli (2025)
Quercetin	Preclinical + Epidemiological	Limited	Low–moderate	Borderline (500–1,000 mg/day)	Limited RCTs	Moderate	Wang et al. (2026)
Resveratrol	Preclinical + Limited human	Limited	Poor	Often unrealistic without metabolic protection (250–500 mg/day)	Rapid metabolism	Low–Moderate (needs formulation)	Singh et al. (2015)
Curcumin	Preclinical	Limited	Very poor	Unrealistic without nanoformulation (80–200 mg/day)	Low systemic exposure	Low (requires delivery optimization)	Lehoczki et al. (2025)
Sulforaphane	Preclinical	Minimal	Moderate	Potentially feasible (10–40 mg/day)	Limited cardiovascular trials	Moderate (early-stage)	Zhao et al. (2025)
Anthocyanins	Preclinical + limited human	Limited	Moderate	Feasible (50–200 mg/day)	Heterogeneous data	Moderate	Salehi et al. (2020)
Saponins (ginsenosides)	Preclinical	Minimal	Low	Unclear (Poor absorption and lack of standardized extracts)	Poor PK data	Low	Juang and Liang (2020)
Phenolic acids	Preclinical	Minimal	Variable	Feasible	Weak clinical evidence	Low–Moderate	Kumar and Goel (2019)
Terpenoids (carotenoids)	Epidemiological	Yes	Good	Feasible (10–30 mg/day)	Indirect evidence	Moderate	Bufka et al. (2024)

### 13.3 Specific recommendations for clinical advancement

#### 13.3.1 Standardized pharmacokinetics and biodistribution

To bridge the “Death Valley” of clinical translation, future investigations must incorporate standardized pharmacokinetic evaluations, including key parameters such as area under the curve (AUC), half-life (T_1_/_2_), and maximum plasma concentration (Cmax), alongside biodistribution studies. These data are essential to confirm adequate myocardial accumulation, thereby enhancing translational relevance (Sleder et al., 2016; Ugariogu et al., 2025).

#### 13.3.2 Validation in large-animal models

Validation in large-animal models, such as porcine or canine systems, is crucial to confirm that cardioprotective effects observed in rodent models can be translated to human-scale cardiovascular physiology. These models more accurately replicate human cardiac structure, hemodynamics, and disease progression, thereby improving the reliability of preclinical findings (Tsang et al., 2016; Osada et al., 2023).

#### 13.3.3 Nano-enablement and formulation optimization

Given the widespread issue of poor bioavailability among natural compounds, formulation optimization is essential prior to clinical advancement. Compounds such as curcumin and berberine should only progress to clinical trials when delivered through enabling platforms, including liposomes or solid lipid nanoparticles (SLNs), which enhance systemic exposure, prolong half-life, and improve tissue targeting (Huang et al., 2024).

#### 13.3.4 Targeting residual cardiovascular risk

Natural bioactive compounds should be evaluated as adjunct therapies alongside standard-of-care treatments, such as statins and antiplatelet agents. In particular, clinical studies should focus on patients with residual inflammatory risk, characterized by elevated C-reactive protein (CRP >2 mg/L), where additional anti-inflammatory or pleiotropic effects may provide meaningful therapeutic benefit (Volpe and Presta, 2018).

### 13.4 Clinical development roadmap for lead natural compounds

#### 13.4.1 Regulatory pathways and standardization

A structured clinical development pathway requires alignment with established regulatory frameworks, such as the FDA Botanical Drug Development Guidance (https://www.fda.gov › search-fda-guidance-documents) or the European Medicines Agency (EMA) guidelines for herbal medicinal products (https://www.ema.europa.eu › multidisciplinary-guidelines). A key consideration is the distinction between standardized botanical extracts and isolated compounds. While standardized extracts may offer synergistic, multi-target effects, they present challenges related to batch-to-batch consistency and regulatory approval (Xiong et al., 2013). In contrast, isolated compounds provide greater consistency and clearer pharmacokinetic characterization but may lack the pleiotropic benefits of complex mixtures (Sasidharan et al., 2011). Both approaches require rigorous chemical characterization, quality control, and reproducible manufacturing processes.

#### 13.4.2 Phase I/II clinical trial design strategy

Early-phase clinical trials should follow a structured and mechanistically driven approach. Phase I studies should prioritize safety, tolerability, pharmacokinetics, and dose-escalation to establish HED, ensuring adequate systemic and myocardial exposure. Phase II trials should focus on efficacy using biomarker-driven endpoints, including inflammatory markers (e.g., CRP), endothelial function, and lipid profiles. For compounds with poor native bioavailability, clinical evaluation should only proceed using optimized formulations such as nano-delivery systems. Additionally, patient stratification is critical; targeting individuals with residual inflammatory risk or conditions such as ischemia with non-obstructive coronary arteries (INOCA) may enhance the likelihood of detecting clinically meaningful effects (Butler et al., 2026).

#### 13.4.3 Prioritization of compounds for clinical advancement

Based on current evidence, pharmacokinetic feasibility, and translational readiness, a tiered clinical development strategy is proposed. In the short term, compounds with existing human data and favorable pharmacokinetics, such as berberine and phytosterols, are suitable for advancement into Phase III adjunctive trials, particularly for lipid management (Salehi B. et al., 2021; Ai et al., 2021). In the mid-term, formulation-dependent candidates such as nano-curcumin and PEGylated berberine should be evaluated in Phase II trials targeting post-myocardial infarction inflammation and ventricular remodeling (Laurindo et al., 2023; Harrison et al., 2021). In the long term, compounds such as sulforaphane and saponins require further Phase I pharmacokinetic and pharmacodynamic studies to validate their mechanisms, including Nrf2-mediated antioxidant effects, before progressing to later-stage trials.

## 14 Molecular mechanisms of phytochemicals in IHD management

The cardioprotective effects of natural products in IHD are mediated through coordinated modulation of interconnected molecular pathways rather than isolated mechanisms. Diverse phytochemical classes, including polyphenols, terpenoids, and alkaloids, converge on key regulatory nodes such as nuclear factor erythroid 2-related factor 2 (Nrf2), NF-κB, and PI3K/Akt pathways, which collectively regulate oxidative stress, inflammation, endothelial function, and cell survival (Syed et al., 2023). Activation of Nrf2 enhances endogenous antioxidant defenses, while inhibition of NF-κB attenuates pro-inflammatory signaling; simultaneously, PI3K/Akt signaling promotes endothelial NO production and reduces apoptosis (Khoi et al., 2024). Importantly, these pathways exhibit significant cross-talk, forming an integrated signaling network that underlies the overlapping cardioprotective effects of structurally diverse natural bioactive compounds (Wardyn et al., 2015) (Table 3).

**TABLE 3:** Mechanistic mapping and translational relevance of natural compounds in IHD.

Compound/class	Representative compounds	Therapeutic Target nodes	Key mechanisms	Reference
Polyphenols	Resveratrol	Oxidative stress/Mitochondria	AMPK/SIRT1 activation, ROS reduction	Yun et al. (2014), Fusi et al. (2018), Guan et al. (2025)
	Quercetin	Oxidative stress/Inflammation	Nrf2 activation, NF-κB inhibition	Minj and Satapathy (2025)
	Catechins (EGCG)	Endothelial dysfunction/Inflammation	eNOS activation, NLRP3 inhibition	Carulli et al. (2025), Liu et al. (2003)
	Anthocyanins	Endothelial protection	Antioxidant, NO enhancement	Liu et al. (2024), Ciumărnean et al. (2020)
Curcuminoids	Curcumin	Inflammation/Oxidative stress	NF-κB inhibition, Nrf2 activation	Minj and Satapathy (2025)
Alkaloids	Berberine	Lipid metabolism	AMPK activation, LDL-c reduction	Srivastava et al. (2012), Liu Y. et al. (2022)
Organosulfur compounds	Allicin (Garlic)	Thrombosis/Endothelial	Antiplatelet, NO enhancement	Seki and Hosono (2025), Wang H. et al. (2025)
Phytosterols	β-sitosterol	Lipid dysregulation	Cholesterol absorption inhibition	Makhmudova et al. (2021), Poli et al. (2021)
Terpenoids	Carotenoids (β-carotene, lycopene)	Oxidative stress	ROS scavenging	Wang M. et al. (2023)
Isothiocyanates	Sulforaphane	Oxidative stress	Nrf2 activation	Kamal et al. (2022)
Flavonoids (broad class)	Apigenin, Luteolin	Inflammation	NF-κB inhibition	Liu et al. (2024), Ciumărnean et al. (2020), Gross (2004)
Saponins	triterpenoid saponins (Ginsenosides)	Multiple (oxidative/inflammatory)	Antioxidant, calcium regulation	Ma et al. (2022), Li Q. et al. (2020)
Phenolic acids	Gallic acid, Caffeic acid	Oxidative stress	Antioxidant activity	Ali et al. (2020), Cheng et al. (2017)
Quinones	-	Mitochondrial function	Electron transport support, antioxidant	Akanda et al. (2025), Li M. et al. (2020)

### 14.1 Antioxidant activity and reduction of oxidative stress

Oxidative stress plays a central role in IHD pathogenesis by promoting endothelial dysfunction, LDL-c oxidation, mitochondrial damage, and myocardial injury. Many phytochemicals exert antioxidant effects through both direct ROS scavenging and activation of endogenous antioxidant systems, including superoxide dismutase (SOD), catalase (CAT), and glutathione peroxidase (GPX).

A key regulatory mechanism involves activation of the Nrf2/ARE signaling pathway, which upregulates antioxidant enzymes such as heme oxygenase-1 (HO-1) and NAD(P)H quinone oxidoreductase-1 (NQO1), thereby reducing oxidative stress–induced endothelial dysfunction and atherosclerotic plaque formation (Arauna et al., 2019; Minj and Satapathy, 2025). In parallel, activation of AMPK/SIRT1/FOXO signaling, particularly by compounds such as resveratrol, enhances mitochondrial function, reduces ROS generation, and improves cellular energy homeostasis (Jeong et al., 2006; Mann et al., 2007; Sofiullah et al., 2023).

Excessive ROS production from NOX contributes to eNOS uncoupling and mitochondrial dysfunction. Polyphenols inhibit NOX activity, thereby reducing superoxide generation in endothelial and vascular smooth muscle cells, limiting LDL-c oxidation, and restoring vascular function. Additionally, phytochemicals preserve mitochondrial redox balance by reducing mitochondrial ROS production and maintaining ATP synthesis, which is critical in limiting ischemia–reperfusion injury. Collectively, these antioxidant mechanisms preserve endothelial integrity, reduce myocardial damage, and slow IHD progression (Hua et al., 2022; Tőzsér and Benkő, 2016).

### 14.2 Anti-inflammatory effects

Chronic vascular inflammation is a key driver of atherosclerosis progression and plaque instability. Phytochemicals suppress inflammatory signaling primarily through inhibition of NF-κB and MAPK pathways, leading to reduced expression of pro-inflammatory cytokines such as TNF-α, IL-6, and IL-1β, as well as adhesion molecules (Andrei et al., 2025). This attenuation of inflammatory signaling decreases endothelial activation and limits leukocyte recruitment to the vascular wall, thereby reducing the initiation and propagation of atherosclerotic lesions.

The NLRP3 inflammasome represents another critical mediator linking metabolic stress to vascular inflammation. Its activation promotes caspase-1–dependent maturation of IL-1β and IL-18, contributing to endothelial dysfunction and myocardial injury. Phytochemicals inhibit NLRP3 inflammasome activation, thereby reducing cytokine release and attenuating inflammatory damage, which ultimately slows plaque progression and enhances vascular stability (Zhang Y. et al., 2024; Yarmohammadi et al., 2022).

In addition to these core pathways, phytochemicals modulate inflammation through several complementary mechanisms. They suppress TLR4/MyD88 signaling, which plays a central role in sterile inflammation following ischemic injury, thereby reducing downstream NF-κB activation and cytokine production (Ebrahimi et al., 2023). Activation of peroxisome proliferator-activated receptor-γ (PPAR-γ) further contributes to anti-inflammatory effects by inhibiting pro-inflammatory gene transcription and reducing macrophage activation (Zhang Y. et al., 2024; Yarmohammadi et al., 2022). Moreover, phytochemicals downregulate endothelial adhesion molecules such as VCAM-1, ICAM-1, and E-selectin, limiting leukocyte adhesion and infiltration into the vascular intima (Ebrahimi et al., 2023). They also influence immune cell dynamics by promoting a shift in macrophage polarization from the pro-inflammatory M1 phenotype toward the anti-inflammatory M2 phenotype, enhancing tissue repair processes and contributing to plaque stabilization (Liu C. et al., 2025). This M1 to M2 macrophage polymerization has been reported with several phytochemicals, including curcumin and resveratrol, which modulate inflammatory signaling and enhance tissue repair (Momtazi-Borojeni et al., 2019; Hedayati et al., 2023).

**FIGURE 7:**
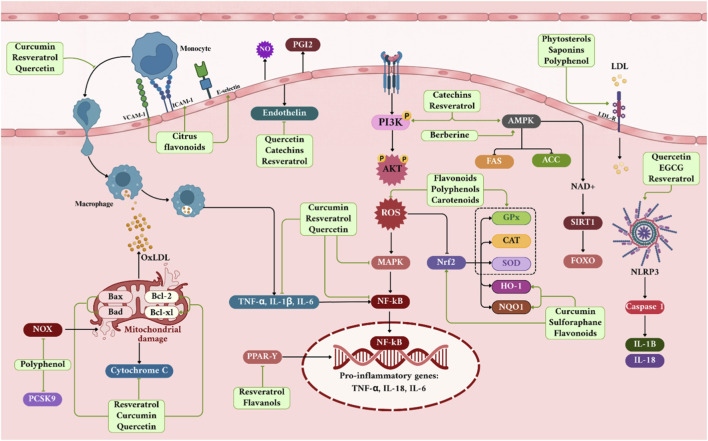
Molecular mechanisms by which polyphenols and related phytochemicals attenuate atherosclerosis and vascular inflammation. This figure summarizes the integrated antioxidant, anti-inflammatory, metabolic, and survival pathways targeted by major phytochemicals in ischemic heart disease. Compounds such as resveratrol, curcumin, quercetin, and catechins reduce oxidative stress by inhibiting NADPH oxidase–derived ROS and activating AMPK/SIRT1/FOXO and Nrf2 signaling, enhancing antioxidant defenses (HO-1, NQO1, SOD, CAT, GPx) and preserving mitochondrial function. They suppress inflammatory cascades through inhibition of NF-κB, MAPK, TLR4, and NLRP3 inflammasome pathways, lowering TNF-α, IL-1β, and IL-6 production. Additional effects include activation of PI3K/Akt-mediated endothelial NO signaling, modulation of lipid metabolism via LDL-cR/PCSK9 pathways, inhibition of endothelin-induced vasoconstriction, and regulation of apoptosis through Bcl-2 family proteins and cytochrome c release.

Collectively, these coordinated actions reduce vascular inflammation, limit immune cell infiltration, stabilize atherosclerotic plaques, and ultimately lower the risk of ischemic events. Figure 7 summarizes these interconnected anti-inflammatory mechanisms.

### 14.3 Improvement of endothelial function

Endothelial dysfunction, characterized by reduced NO bioavailability and impaired vasodilation, represents a central feature in the pathogenesis of IHD. Phytochemicals enhance endothelial function primarily through activation of the PI3K/Akt/eNOS signaling pathway, which increases NO production and promotes vascular relaxation (Liu C. et al., 2025; Vilahur et al., 2015; de Paula Silva et al., 2022). At the same time, their antioxidant effects reduce oxidative stress–mediated NO degradation, thereby restoring endothelial signaling balance and improving vascular responsiveness.

In addition, phytochemicals suppress the expression of endothelin-1 (ET-1), a potent vasoconstrictor, which further contributes to improved vascular tone and reduced coronary vasospasm (Carulli et al., 2025; Liu et al., 2003). Beyond these mechanisms, they support endothelial repair processes by enhancing the mobilization and activity of EPCs, facilitating vascular regeneration and neovascularization. They also contribute to the preservation of the endothelial glycocalyx, a critical structure involved in mechanotransduction and vascular barrier integrity, thereby maintaining normal endothelial function under hemodynamic stress (Carulli et al., 2025; Franceković and Gliemann, 2023; Wan et al., 2024).

Collectively, these mechanisms improve coronary perfusion, reduce vascular stiffness, and restore microvascular function, highlighting the central role of phytochemicals in targeting endothelial dysfunction in IHD.

### 14.4 Lipid-lowering and anti-atherogenic effects

Dyslipidemia is a major contributor to atherosclerosis and the progression of IHD, primarily through promoting lipid accumulation, foam cell formation, and plaque development. Phytochemicals modulate lipid metabolism through multiple complementary mechanisms, including inhibition of intestinal cholesterol absorption, enhancement of LDL-c receptor expression, and reduction of hepatic cholesterol synthesis, collectively leading to decreased LDL-c levels and improved lipid profiles (Scavuzzi and Dichi, 2025; Wei et al., 2025).

A central mechanism underlying these effects is the activation of AMPK, which inhibits key lipogenic enzymes such as acetyl-CoA carboxylase (ACC) and fatty acid synthase (FAS), while simultaneously promoting fatty acid oxidation. This metabolic shift reduces circulating triglyceride levels and limits lipid accumulation within vascular tissues, thereby attenuating atherogenesis (Srivastava et al., 2012; Liu Y. et al., 2022).

In addition, phytochemicals exert anti-atherogenic effects through suppression of PCSK9, which enhances LDL-c receptor availability and promotes cholesterol clearance (Liou et al., 2024). They also facilitate reverse cholesterol transport by upregulating ATP-binding cassette transporters ATP-binding cassette transporter A1 (ABCA1) and ATP-binding cassette subfamily G member 1 (ABCG1) in macrophages, promoting cholesterol efflux and reducing foam cell formation (Wang J. et al., 2022; Arshad et al., 2025). Furthermore, their antioxidant properties reduce the formation of oxidized LDL (oxLDL), thereby limiting macrophage uptake and plaque progression (Maheshwari, 2020). Some compounds also modulate bile acid metabolism via farnesoid-X-receptor (FXR) and cytochrome P450 Family 7 Subfamily A Member 1 (CYP7A1) signaling, enhancing cholesterol conversion and excretion (Liu J. et al., 2020).

Collectively, these mechanisms contribute to reduced lipid accumulation, slowed plaque formation, and stabilization of existing atherosclerotic lesions.

### 14.5 Antithrombotic and antiplatelet actions

Thrombus formation following plaque rupture is a critical event in acute ischemic syndromes, and phytochemicals exert significant antithrombotic effects by targeting both platelet activation and the coagulation cascade. These compounds inhibit platelet aggregation, reduce thromboxane A_2_ synthesis, and interfere with key intracellular signaling pathways, including PI3K/Akt, MAPK, and calcium mobilization, thereby attenuating platelet activation, granule secretion, and thrombus formation (Syed et al., 2023; Shafiekhani et al., 2016; Hosseini et al., 2025; Tamer et al., 2022). In addition to directly modulating platelet function, phytochemicals promote an antithrombotic endothelial phenotype by enhancing the release of NO and prostacyclin (PGI_2_), both of which inhibit platelet adhesion and aggregation at sites of vascular injury and contribute to improved coronary blood flow. Further antithrombotic effects are mediated through inhibition of coagulation factors such as thrombin (factor IIa) and factor Xa, as well as reduction of tissue factor (TF) expression, which limits activation of the extrinsic coagulation pathway. Moreover, phytochemicals suppress platelet–leukocyte interactions through downregulation of adhesion molecules such as P-selectin, thereby reducing thromboinflammatory responses and contributing to plaque stabilization (Tamer et al., 2022). Collectively, these mechanisms reduce thrombus formation and enhance vascular stability in IHD.

### 14.6 Anti-apoptotic and cardioprotective effects

Ischemia–reperfusion injury induces cardiomyocyte apoptosis through mitochondrial dysfunction, oxidative stress, calcium overload, and activation of intrinsic apoptotic signaling pathways. Phytochemicals counteract these processes by activating pro-survival signaling cascades such as PI3K/Akt and AMPK, which enhance cellular survival, improve energy metabolism, and reduce oxidative damage (Yun et al., 2014).

At the mitochondrial level, these compounds regulate apoptotic signaling by increasing the expression of anti-apoptotic proteins such as B-cell lymphoma 2 (Bcl-2) and B-cell lymphoma-extra-large (Bcl-xL), while decreasing pro-apoptotic proteins including Bcl-2–associated X protein (Bax) and Bcl-2–associated agonist of cell death (Bad). This shift in the balance of Bcl-2 family proteins prevents mitochondrial membrane permeabilization and cytochrome c release. In parallel, phytochemicals inhibit the activation of key executioner caspases, particularly caspase-3 and caspase-9, thereby limiting DNA fragmentation and cardiomyocyte death (Lin et al., 2016).

Additionally, phytochemicals modulate the interplay between autophagy and apoptosis by promoting protective autophagy while preventing excessive or maladaptive cell death. This coordinated regulation supports cellular homeostasis and enhances myocardial resilience during ischemic stress. Overall, these mechanisms preserve myocardial viability, reduce infarct size, and improve functional recovery following ischemic injury (Dong et al., 2025).

## 15 Formulation strategies for IHD therapeutics

**FIGURE 8:**
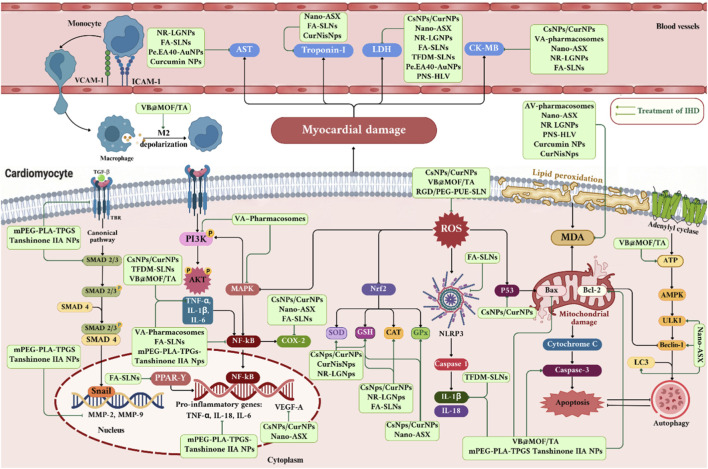
Schematic illustration of nanoparticle-mediated cardioprotection mechanisms in IHD. This figure depicts how diverse nanoformulations—including lipid nanoparticles, solid lipid nanoparticles (SLNs), liposomes, lipid–polymer hybrids, metal–organic framework systems, polymeric nanoparticles, and targeted RGD- or mitochondria-modified carriers—enhance myocardial drug delivery and therapeutic efficacy in IHD. By optimizing physicochemical properties (size, PDI, zeta potential, drug loading, entrapment efficiency, and controlled release), these systems improve cardiac accumulation, bioavailability, and sustained drug action compared with free compounds. At the biochemical level, nanoformulations reduce cardiac injury markers (CK-MB, LDH, troponin-I, AST/ALT), suppress lipid peroxidation (MDA), and restore antioxidant defenses (SOD, CAT, GPx, GSH). Molecularly, they inhibit ROS generation, NF-κB, MAPK, NLRP3 inflammasome, COX-2, and pro-inflammatory cytokines (TNF-α, IL-1β, IL-6, IL-18), while modulating PI3K/Akt, AMPK, PPAR-γ, and Nrf2 pathways. They also regulate apoptosis (↓Bax, ↓caspase-3, ↑Bcl-2), enhance autophagy (Beclin-1, ULK1, LC3), promote mitochondrial protection and ATP preservation, and attenuate fibrosis and remodeling.

Nanotechnology transforms drug delivery for cardiovascular diseases by enabling targeted myocardial delivery, sustained release, and improved bioavailability. Unlike conventional oral or intravenous drugs, which often suffer from rapid clearance, poor solubility, and off-target effects, nanoformulations such as lipid–polymer hybrids, liposomes, and polymeric nanoparticles enhance cardiac accumulation and enable precise therapeutic delivery (Jha et al., 2024) (Table 4; Figure 8).

**TABLE 4:** Nanoparticle-based formulations for IHD.

Nanoparticles	Preparation method	Particle size - PDI	Zeta potential (mV)	Drug loading %	Entrapment efficacy %	References
Chitosan nanoparticles (CsNPs)	NA	35.63 nm - NA	NA	NA	NA	Mosa et al. (2021)
Curcumin nanoparticles (CurNPs)	NA	40 nm - NA	NA	NA	NA	
VB@MOF/TA nanoparticles	Electrostatic adsorption of VB onto MOF nanoparticles and TA conjugation onto VB@MOF surface	∼350 nm - NA	−29.5	NA	∼62%	Li et al. (2025)
Vanillic acid pharmacosomes	Refluxing method followed by thin film hydration	229.7 nm −0.29	−30.8	NA	NA	Dawoud et al. (2023)
Nano-astaxanthin NLCs	Hot high-pressure homogenization	∼143 nm -**<0.3**	∼−30	NA	∼92%	Radwan et al. (2025)
Naringin lignin NPs	Modified phase separation	∼138 nm - 0.256	∼−27	∼15.72%	∼92%	Jaiswal et al. (2023)
TFDM SLNs	High-shear homogenization followed by ultrasonication	∼105 nm - 0.21	−28.7	8.61%	Luteolin: 83.98% Rosmarinic acid: 87.01% Tilianin: 88.82%	Tan et al. (2017)
Ferulic acid SLNs	Nano-template engineering	∼77 nm −0.14	−12.7	NA	NA	Ishtiaq et al. (2023)
Ginger extract PU NPs	Interfacial polyaddition combined with spontaneous emulsification	∼90 nm - 0.7	+25 to +28	NA	∼82.9	Borcan et al. (2019)
Gold NPs (Pe.EA40-AuNPs)	Green synthesis	∼203 nm - 0.32	∼−14	NA	NA	Ibrar et al. (2018)
Curcumin nanoparticles (nC)	NA	30–100 nm - NA	NA	NA	NA	Boarescu et al. (2019)
Curcumin–nisin PLA NPs	Double emulsion-diffusion-evaporation method	284 nm -NA	NA	35%	NA	Nabofa et al. (2018)
PNS-HLV	double-emulsion solvent evaporation and thin-film hydration	338 nm -NA	−44.7	NA	R1: 57.5 Rb1: 83.1 Rg1: 40.5	Zhang et al. (2012)
PEGylated berberine liposomes	Ethanol injection method	110 nm - 0.048	NA	0.3 mg/mL	10.4	Allijn et al. (2017)
RGD/PEG-PUE-SLN	Modified solvent evaporation	110.5 nm - 0.23	−26.2	16.5	85.7	Dong et al. (2017)
RGD-S/P-LPNs	Nanoprecipitation	139.5 nm - 0.16	−32.4	Sal B: 3.2 PNS: 4.3	Sal B:90.3 PNS: 89.1	Qiu et al. (2017)
TPP-TPGS/TN/LPNs	Nanoprecipitation	∼140 nm - 0.16	∼-10	NA	∼90%	Qiu et al. (2017)
mPEG-PLA-TPGS polymeric Tan IIA nanoparticles	Thin film hydration method	100–200 nm - PDI <0.25	∼-7.12	1.48	61.30	Qiu et al. (2017)

Physicochemical parameters—particle size, polydispersity index (PDI), surface charge (zeta potential), drug loading (DL), entrapment efficiency (EE), and release kinetics govern the quality and therapeutic performance of nanoparticles in cardiovascular applications, particularly IHD and CAD, by controlling stability, bioavailability, targeting efficiency, and myocardial drug retention. Particle sizes of 10–200 nm optimize vascular permeability, cellular uptake, and renal clearance, whereas oversized or aggregated particles impair penetration and targeting. PDI below 0.3 ensures monodisperse, reproducible formulations with consistent biodistribution, while values above 0.3–0.4 increase heterogeneity, aggregation, destabilized release, and reduced myocardial targeting. Drug loading (DL, % w/w) remains key for therapeutic efficiency and translational feasibility. Most nanoparticles exhibit low DL (<10 wt%), but higher loading reduces carrier burden and dosing frequency despite formulation challenges. Entrapment efficiency (EE), inconsistently defined, plays an essential role in minimizing drug loss and ensuring robustness. Release kinetics prove critical in high-DL systems, as burst or uncontrolled release due to weak drug–carrier interactions or open pores causes subtherapeutic exposure or toxicity. Strategies such as surface functionalization, stimuli-responsive gatekeepers, and core–shell designs enable sustained or triggered release, extend half-life to hours or days, and support prolonged myocardial drug action and improved IHD outcomes (Li et al., 2023; Liu Y. et al., 2020; Danaei et al., 2018).

Nanoformulations outperform traditional therapies in IHD by superiorly modulating biochemical, molecular, and genetic factors for greater pathological normalization in preclinical models. Biochemically, they more effectively reduce cardiac injury markers creatine kinase-MB (CK-MB), lactate dehydrogenase (LDH), cardiac troponin I (cTnI) and oxidative stress indicators such as malondialdehyde (MDA), lipid peroxidation (LPO), thiobarbituric acid reactive substances (TBARS), and NO, while robustly restoring antioxidants: SOD, CAT, GPX, glutathione S-transferase (GST), reduced glutathione (GSH), and total antioxidant capacity (TAC). Molecularly, nanoformulations potently suppress pro-inflammatory cytokines and mediators TNF-α, IL-1/IL-6/IL-18, cyclooxygenase-2 (COX-2), NF-κB, NLRP3 inflammasome, and p-JNK—upregulate PPAR-γ, and in apoptosis pathways, optimize Bax (pro-apoptotic)/Bcl-2 (anti-apoptotic) ratios while reducing cleaved caspase-3 and p53 more than free drugs. Genetically and via autophagy, they upregulate repair genes Beclin-1, Unc-51 like autophagy activating kinase 1 (ULK1), and microtubule-associated protein 1 light chain 3 beta (LC3B); suppress microRNA-217 (miR-217); normalize VEGF-A, atrial natriuretic factor (ANF), TGF-β1, Smad3, and matrix metalloproteinases (MMP-2/9); and improve lipid profiles (High-density lipoprotein cholesterol (HDL-c), LDL-c, very low-density lipoprotein cholesterol (vLDL-c)) and liver enzymes; alanine aminotransferase (ALT) and aspartate aminotransferase (AST), with histopathological validation confirming targeted cardioprotection superiority.

Notably, the majority of nanoformulations discussed in this section are derived from the same natural bioactive compounds highlighted in Section 12, including polyphenols (e.g., naringin and ferulic acid), curcuminoids (curcumin), alkaloids (berberine), carotenoids (astaxanthin), and saponins (e.g., puerarin-related compounds). These compounds exhibit well-established cardioprotective mechanisms such as antioxidant, anti-inflammatory, anti-apoptotic, and endothelial-protective effects yet their clinical translation is often limited by poor aqueous solubility, low bioavailability, rapid systemic clearance, and inadequate tissue targeting.

The nanoformulation strategies described herein such as curcumin nanoparticles, naringin-loaded nanoparticles, ferulic acid solid lipid nanoparticles, and liposomal berberine illustrate how these limitations can be overcome. Specifically, these systems enhance pharmacokinetic profiles, improve myocardial accumulation, enable controlled and sustained release, and facilitate targeted delivery to ischemic tissues, as reflected in the studies discussed above, resulting in superior cardioprotective outcomes compared to their free-drug counterparts.

Accordingly, nano-delivery systems should be considered enabling platforms that potentiate the therapeutic efficacy of natural compounds, rather than independent interventions, thereby reinforcing their role within integrative strategies for ischemic heart disease management.

A recent study reports chitosan nanoparticles (CsNPs, 35.63 nm, spherical) and curcumin nanoparticles (CurNPs, 40 nm, crystalline). The nanoparticles are tested *in vivo* in Wistar male rats and demonstrate cardioprotective effects by restoring redox balance, improving cardiac enzymes (CK-MB, LDH), normalizing lipid profiles (↑HDL-c, ↓LDL-c, ↓vLDL-c, triglyceride [↓TAG]), enhancing antioxidant defenses (GPX, GST, CAT, SOD, GSH, TAC), reducing oxidative stress markers (↓TBARS, ↓LPO, ↓NO), downregulating apoptotic and inflammatory markers (p53, TNF-α, IL-6), and reducing expression of VEGF-A, COX-2, and ANF; however, the absence of a direct comparison with free (non-nano) chitosan or curcumin limits the ability to determine whether these nanoformulations confer superior cardioprotection beyond their free-drug counterparts (Mosa et al., 2021).

A study demonstrates the preparation of vitamin B-loaded metal–organic framework/tannic acid (VB@MOF/TA) nanoparticles via electrostatic adsorption of vitamin B (VB) onto metal-organic framework (MOF) nanoparticles, followed by tannic acid (TA) conjugation to the VB@MOF surface. The resulting nanoparticles (∼350 nm, −29.5 mV) feature a metal–organic framework core loaded with verbascoside at a 2:1 w/w ratio (∼62% encapsulation efficiency) and are surface-functionalized with tannic acid. In *in vitro* studies, the nanoparticles were efficiently internalized, escape lysosomes, target mitochondria, scavenge ROS, reduce mitochondrial stress, restore membrane potential, increase ATP, and regulate apoptotic proteins (Bax↓, C-caspase 3↓, Bcl-2↑). TA exhibits multi-enzyme mimetic activity (SOD-, CAT-, peroxidase [POD]-like), mitigates DNA damage, suppresses inflammatory cytokines (TNF-α, IL-1β, IL-6), elevates IL-10, promotes M2 macrophage polarization, and enhances cell survival. *In vivo*, VB@MOF/TA improves cardiac function, promotes angiogenesis (cluster of differentiation 31^+^ [CD31^+^], alpha-smooth muscle Actin [α-SMA^+^]), reduces infarct size, and attenuates ventricular remodeling, with direct comparison to free VB showing superior ROS scavenging, mitochondrial protection, anti-apoptotic effects, and a 7.72% improvement in left ventricular ejection fraction, confirming meaningful advancement in cardioprotection over the free drug (Li et al., 2025).

A study develops vanillic acid (VA)-loaded pharmacosomes (VA–phosphatidylcholine, 229.7 nm, PDI 0.29, −30.8 mV, EE ∼97%) to overcome poor solubility and low oral bioavailability (∼25%). *In vivo*, the pharmacosome formulation markedly enhances cardioprotection compared to free VA, increases relative bioavailability by 384%, elevates C_max_ (173.72 vs. 132.65 μg/mL), and extends mean residence time threefold (1747.7 vs. 572 min). Functionally, serum CK-MB is normalized to 244.0 ± 56.8 U/L versus 466.5 ± 25.1 U/L with free VA, while cardiac GSH rises to 7.05 ± 0.3 μmol/g compared to 3.90 ± 0.4 μmol/g. At the molecular level, the pharmacosome suppresses miR-217 by 45.3% (non-significant change with free VA), reduces MAPK by 45.1% (vs. 29.8%), and fully normalizes PI3K expression (vs. 33.2% reduction). Inflammatory markers are also better controlled, with IL-6 decreases by 62.3% (vs. 46.5%) and TNF-α returns to normal levels (vs. 54.1% reduction). Histopathology and electrocardiography confirm preserved myocardial structure and function, collectively demonstrating that VA pharmacosomes substantially advance cardioprotection beyond the free drug (Dawoud et al., 2023).

A study investigates nano-formulated astaxanthin (nano-ASX) as nanostructured lipid carriers (nanostructured lipid carriers [NLCs], 143 nm, PDI <0.3, −30 mV, EE ∼92%) prepared via hot high-pressure homogenization. *In vivo*, nano-ASX reduces serum CK-MB, LDH, and troponin-I, normalizes liver and kidney function, attenuates myocardial LPO and NO, restores antioxidants (GSH, GPX, glutathione reductase), suppresses COX-2 and VEGF, and activates autophagy-related genes (Beclin-1, ULK1, LC3B), promoting cellular repair and mitochondrial homeostasis. Compared to free ASX, nano-ASX demonstrates superior cardioprotection, with greater reductions in CK-MB (60.4% vs. 34.2%) and troponin-I (78.2% vs. 65.8%), enhanced antioxidant restoration (GSH +103.9% vs. +73.7%), stronger suppression of COX-2 (30.3% vs. 22.7%) and VEGF (37.5% vs. 26.7%), superior upregulation of autophagy genes, and near-normal AST and urea, while histopathology confirms mild to moderate myocardial injury versus moderate damage with free ASX (Radwan et al., 2025).

A study focuses on lignin-based naringin-loaded nanoparticles (NR-LGNPs, 138 nm, PDI 0.256, −27 mV, EE ∼92%, DL 15.72%, ∼89% drug release over 24 h) prepared via a modified phase-separation technique. *In vivo*, intravenous NR-LGNPs (10 mg/kg) attenuate myocardial injury by reducing CK-MB, LDH, AST, and ALT, restoring antioxidants (SOD, CAT, GSH), decreasing LPO (MDA, TBARS), improving lipid profiles, normalizing systolic blood pressure, and correcting electrocardiographic abnormalities, while histopathology and 2,3,5-triphenyltetrazolium chloride (TTC) staining confirm preserved myocardial architecture and reduced infarct size. Compared to free naringin, NR-LGNPs demonstrate superior cardioprotection: ALT and AST are restored to control levels, CK-MB and LDH show greater improvement, infarct size decreases from 21.77% ± 3.32% with free naringin to 11.41% ± 1.61%, antioxidants are more fully restored, TBARS is further reduced, total cholesterol and triglycerides normalize, HDL improves, PR and QT intervals and ST segments are corrected more effectively, and systolic blood pressure rises to near-normal levels (121.74 ± 4.51 mmHg vs. 112.27 ± 5.60 mmHg with free naringin), collectively confirming that NR-LGNPs meaningfully advance cardioprotection beyond the free drug (Jaiswal et al., 2023).

This study develops solid lipid nanoparticles encapsulating total flavonoid extract from Dracocephalum moldavica L. (TFDM-SLNs) using Compritol® 888 ATO with soy lecithin and Tween-80 via high-shear homogenization and ultrasonication. The nanoparticles are spherical (∼105 nm, PDI ≈0.21, −28.7 mV) with high entrapment efficiencies (luteolin ∼84%, rosmarinic acid ∼87%, tilianin ∼89%), DL of 8.61%, and sustained release (∼96% over 48 h). *In vivo*, oral TFDM-SLNs reduce infarct size, lower LDH and CK, suppress IL-1β and TNF-α, and improve myocardial histopathology. Compared to free TFDM in a myocardial ischemia–reperfusion model, TFDM-SLNs demonstrate superior cardioprotection, where both treatments significantly improve outcomes versus the model (*P* < 0.001), but the nanoparticle formulation achieves lower LDH and CK levels and greater reductions in IL-1β and TNF-α (*P* < 0.001), along with a more pronounced reduction in infarct size, confirming enhanced efficacy over the free drug (Tan et al., 2017).

Ferulic acid–loaded solid lipid nanoparticles (FA-SLNs) are developed via a nano-template engineering approach using stearyl alcohol with poloxamer-188 and Tween-80. The nanoparticles are uniform (∼77 nm, PDI ≈0.14, −12.7 mV). *In vivo*, FA-SLNs attenuate cardiac hypertrophy, reduce infarct size, normalize electrocardiographic changes, lower cardiac biomarkers (cTnI, CK-MB, LDH, ALT, AST), restore antioxidants (GSH, GST, CAT), suppress TBARS, upregulate PPAR-γ, and inhibit inflammatory signaling (NLRP3, NF-κB, p-JNK, COX-2, TNF-α), with histopathology confirming preserved myocardial structure. Compared to free ferulic acid, FA-SLNs demonstrate superior cardioprotection, reducing infarct size from 62.89% (ISO) to 32.19% (free FA) and further to 27.18%, and lowering the heart weight/body weight ratio from 0.477% to 0.4% (free FA) and 0.371%. Both treatments significantly reduce cTnI and CK-MB, but FA-SLNs show stronger effects, with more robust normalization of ALT, AST, LDH, and creatine phosphokinase (CPK), as well as greater improvement in lipid profile parameters, confirming enhanced efficacy over the free drug (Ishtiaq et al., 2023).

The study develops polyurethane (PU) hollow nanoparticles encapsulating ginger extract (GE) via catalyst-free interfacial polyaddition. The nanoparticles have a particle size of ∼90 nm, PDI of 0.7, zeta potential ≈28 mV, encapsulation efficiency ∼82.9%, thermal stability ∼280 °C, and show delayed sustained release. *In vivo*, they reduce transepidermal water loss and erythema compared with free GE, indicating improved biocompatibility and safety. No direct quantitative comparison demonstrates that the nanoformulation advances cardioprotection beyond free GE, as no *in vivo* cardioprotective endpoints such as cardiac biomarkers, infarct size, oxidative stress, or inflammation are evaluated, limiting conclusions regarding therapeutic superiority (Borcan et al., 2019).

This study develops Paeonia emodi ethyl acetate subfraction–derived gold nanoparticles (Pe.EA40-AuNPs) via green synthesis. The nanoparticles are spherical (∼203 nm, PDI 0.32, zeta potential ≈ −14 mV) and stable under physiological conditions. *In vivo*, Pe.EA40-AuNPs (40 mg/kg) reduce ALT, AST, CPK, and LDH, improve lipid profile (↓total cholesterol, triglycerides, LDL-c; ↑HDL), lower atherogenic index, reduce DNA fragmentation, and preserve cardiac histoarchitecture. Compared to free Pe.EA40, the nanoparticle formulation shows improved efficacy at half the dose (40 mg/kg vs. 80 mg/kg), reducing ALT (66.07–60.74; ∼8% reduction), AST (77.08–75.47; ∼2% reduction), CPK (84.86–80.48; ∼5% reduction), and LDH (265.34–247.54 IU/L; ∼7% reduction). Lipid profile is further improved, with total cholesterol reduced from 120.35 to 109.52 mg/dL (∼9%), triglycerides from 130.72 to 115.45 mg/dL (∼12% reduction), LDL-c from 58.54 to 49.97 mg/dL (∼15% reduction), and HDL increased from 28.45 to 32.93 mg/dL (∼16% increase), demonstrating ∼5–15% improvements and comparable or superior protection at 50% lower dose (Ibrar et al., 2018).

The study investigates curcumin nanoparticles (nC, 30–100 nm) to enhance bioavailability and myocardial delivery. *In vivo*, nC normalizes electrocardiographic indices (RR interval, heart rate, QRS width, QT/QTc intervals, ST-segment depression, and T-wave inversion), reduces serum markers (LDH, AST, ALT, glycaemia), attenuates oxidative stress (↓MDA, ↓NO, ↓total oxidative status; ↑thiol content, ↑total antioxidant capacity), and improves histopathology by reducing necrosis, edema, and inflammation. Compared to conventional curcumin (Cs), nC shows superior cardioprotection, improving RR interval (248 vs. 219 ms) and heart rate (242 vs. 274 b/min), preventing QRS prolongation (35 vs. 39 ms), and reducing QT interval (82 vs. 98 ms; ∼16.3% improvement). nC reduces LDH by ∼50% versus MI control and shows significantly better effects than Cs (p < 0.009), while oxidative stress is further improved with MDA levels near control and stronger preservation of total antioxidant capacity, with the 200 mg/kg dose bringing most parameters close to normal (Boarescu et al., 2019).

This study demonstrates curcumin–nisin poly (lactic acid) nanoparticles (CurNisNp, ∼284 nm, DL 35.0%) via double-emulsion diffusion–evaporation, encapsulating curcumin and nisin within a biodegradable PLA matrix. *In vivo*, CurNisNp preserves electrocardiographic parameters, reduces hypertrophy, maintains myocardial structure, lowers oxidative stress (↓hydrogen peroxide, ↓MDA), restores antioxidants (SOD, GSH, GST), reduces inflammatory infiltration and myeloperoxidase activity, and downregulates cTnI and kidney injury molecule-1 (KIM-1). Compared to free curcumin, CurNisNp (21 mg/kg ≈ 60 mg/kg free curcumin) shows superior efficacy, reducing MDA from 0.78 ± 0.06 to 0.18 ± 0.01 μmol/g (∼77% reduction), increasing total thiols from 33.74 ± 3.69 to 48.71 ± 7.99 nmol/mg (∼44% increase), and enhancing SOD from 11.16 ± 1.17 to 16.32 ± 2.28 units/mg (∼46% increase), while also significantly lowering cTnI and preventing atrial fibrillation, demonstrating improved cardioprotection at a lower equivalent dose (Nabofa et al., 2018).

The study prepares Panax notoginsenoside–loaded core–shell hybrid liposomal vesicles (PNS-HLV) by integrating mPEG-PLGA nanoparticle core with a phospholipid liposomal shell via double-emulsion solvent evaporation and thin-film hydration, achieving synchronous and improved entrapment of the principal active saponins notoginsenoside R1 (57.5%), ginsenoside Rb1 (83.1%), and ginsenoside Rg1 (40.5%). *In vitro*, the uniform spherical vesicles with a particle size of approximately 338 nm and strongly negative zeta potential (∼–44.7 mV) exhibit favorable colloidal stability and sustained, pH-dependent drug release with minimal burst effect. *In vivo*, pretreatment reduces infarct size, improves electrocardiographic parameters, and attenuates oxidative stress (↓MDA, ↓H_2_O_2_, restored SOD activity, ↓serum LDH), indicating enhanced cardioprotective efficacy. Compared to PNS solution, nanoparticles, and liposomes, PNS-HLV shows superior performance, increasing Rg1 encapsulation from 15.2% (NP) and 28.3% (LP) to 40.5% (∼2.7-fold and ∼1.4-fold. Drug release is lower (R1: 50.1% vs. 83.9%, Rb1: 34.6% vs. 54.2%, Rg1: 63.4% vs. 90.2%), while *in vivo* LDH decreases from 6,224.5 to 4,022.3 U/L (∼35.4%) versus 5,224.7 U/L with solution, SOD increases from 11.4 to 38.0 U/mgprot (∼233%), and H_2_O_2_ decreases from 5.45 to 3.12 mmol/L (∼42.8%), demonstrating superior cardioprotection (Zhang et al., 2012).

This study demonstrates long-circulating liposome-encapsulated berberine (BB-lip) via ethanol injection using dipalmitoylphosphatidylcholine (DPPC), cholesterol, and 1,2-Distearoyl-sn-glycero-3-phosphoethanolamine–Poly (ethylene glycol) 2000 (DSPE-PEG2000), achieving an entrapment efficacy of 10.4%. The PEGylated liposomes are approximately 110 nm in size, with low polydispersity (PDI ∼0.048), uniform spherical morphology, stable berberine loading (∼0.3 mg/mL), long-term colloidal stability, and minimal premature release. *In vitro*, BB-lip demonstrates controlled release, limiting immediate uptake, while free berberine inhibits LPS-induced IL-6 secretion in macrophages. *In vivo*, intravenous BB-lip preferentially accumulates in infarcted myocardium, partially colocalizes with macrophages, preserves left ventricular ejection fraction and fractional shortening, and reduces ventricular remodeling. Compared to free berberine, BB-lip significantly improves cardiac function, preserving LVEF at 29.5% ± 1.9% versus 18.2% ± 3.2% (∼64% improvement) and fractional shortening at 13.79% ± 0.78% versus 7.97% ± 1.85%. The formulation is effective at 1.5 mg/kg, representing 3 to 66 times lower dose than conventional administration, demonstrating enhanced therapeutic availability and cardioprotection (Allijn et al., 2017).

RGD-modified and PEGylated solid lipid nanoparticles encapsulating puerarin (RGD/PEG-PUE-SLN), prepared by a modified solvent evaporation method, exhibit a particle size of 110.5 nm, a PDI of 0.23, a negative zeta potential (−26.2 mV), high encapsulation efficiency (85.7%), a DL of 16.5%, and sustained drug release. *In vivo*, this formulation shows prolonged circulation (T½ = 2.62 h; AUC = 176.5 μg/mL·h) and preferential accumulation in ischemic myocardium (23.1-fold higher than free puerarin) via passive (EPR) and active (αvβ3 integrin–RGD) targeting, resulting in enhanced antioxidant activity, attenuation of ROS generation, calcium overload, mitochondrial damage, necrosis, and apoptosis, and marked infarct size reduction in an AMI rat model. Compared to free puerarin, RGD/PEG-PUE-SLN significantly improves pharmacokinetics and cardioprotection. The AUC increases from 52.93 to 176.5 μg/mL·h (∼3.3-fold), and T_1_/_2_ extends from 0.73 to 2.62 h (∼3.6-fold). In infarcted heart tissue, the nanoparticle achieves a 23.1-fold higher puerarin concentration versus free drug, 2.2-fold versus non-targeted PUE-SLN, and 1.3-fold versus PEGylated nanoparticles without RGD. Infarct size is reduced to 6.2% compared to 18.1% (PEG-PUE-SLN), 28.3% (PUE-SLN), and 36.0% (free puerarin), representing an 83% reduction versus free drug and a 66% reduction versus non-PEGylated nanoparticles, demonstrating the superior cardioprotective effect of dual-targeting (Dong et al., 2017). Similarly, αvβ3 integrin–targeted lipid–polymer hybrid nanoparticles co-loading salvianolic acid B and Panax notoginsenoside (RGD-S/P-LPNs), prepared by nanoprecipitation, are distinguished by their PLGA core, phospholipid monolayer, and PEGylated lipid shell functionalized with an RGD peptide. They exhibit particle sizes of approximately 139.5 nm with a PDI of 0.16, a negative zeta potential of −32.4 mV, high DL (3.2% for Sal B and 4.3% for PNS), and high entrapment efficiencies (∼90.8% for Sal B and 89.2% for PNS), along with sustained dual-drug release. *In vivo*, RGD-S/P-LPNs demonstrate prolonged circulation, increased AUC, enhanced myocardial drug delivery, and significantly reduced infarct size, outperforming single-drug and non-targeted formulations through confirmed synergistic cardioprotective efficacy in acute myocardial ischemia. Compared with free drugs, the nanoparticles dramatically improve pharmacokinetics: AUC increases from 17.81 to 176.29 mg/L·h for Sal B (∼9.9-fold) and from 18.71 to 213.22 mg/L·h for PNS (∼11.4-fold), while T_1_/_2_ extends from 0.86 to 3.68 h. Targeted delivery leads to significantly higher drug accumulation in the heart for up to 72 h. Most importantly, infarct size is reduced to 26%, versus 48%–53% for free drug groups, representing a 48%–51% reduction relative to free drugs, demonstrating that RGD-S/P-LPNs meaningfully advance cardioprotection through dual-drug synergy, sustained release, PEGylation, and active αvβ_3_ integrin targeting (Qiu et al., 2017).

Tanshinone IIA nanoparticles are developed using two distinct formulations to enhance solubility, myocardial delivery, and cardioprotection. One study develops mPEG-PLA-TPGS polymeric tanshinone IIA nanoparticles using the thin-film hydration method, producing particles with a size of approximately 100–200 nm, a PDI of 0.25, a zeta potential of about −7.12 mV, a DL of 1.48%, and an EE of 61.30%. *In vivo* intravenous administration preserves left ventricular function, limits infarct expansion and ventricular dilation, suppresses cardiomyocyte apoptosis through decreased Bax and cleaved caspase-3 and increased Bcl-2 expression, attenuates inflammation by reducing TNF-α, IL-1β, IL-6, monocyte chemoattractant protein-1 (MCP-1), and IL-18 via inhibition of inhibitor of kappa B (IκB) phosphorylation and NF-κB signaling, and mitigates cardiac fibrosis through downregulation of TGF-β1, Smad3, MMP-2, and MMP-9 and Compared to free tanshinone IIA, the nanoparticle formulation shows superior efficacy at 1 mg/kg versus ineffective free drug even at 10 mg/kg (∼10-fold lower dose), reducing heart weight/body weight ratio by 16%, scar circumference by 21%, and fibrosis by 32% (*p* < 0.05). Apoptosis markers decrease (Bax 0.65-fold, caspase-3 0.63-fold) while Bcl-2 increases 7.8-fold, demonstrating enhanced cardioprotection. A second study fabricates mitochondria-targeted TPP-TPGS/TN lipid–polymer hybrid nanoparticles using the nanoprecipitation method, yielding nanoparticles with a mean size of approximately 140 nm, a PDI of 0.16, a zeta potential of about −10 mV, and an EE of approximately 90%. These mitochondria-targeted nanoparticles enhance cellular uptake, prolong intracellular retention, reduce cytotoxicity, and increase cardiac and mitochondrial accumulation, resulting in superior infarct size reduction and cardioprotection compared with non-modified nanoparticles and free tanshinone IIA and Compared to free tanshinone IIA, TPP-TPGS/TN/LPNs increase AUC to 129.27 mg/L·h versus 5.49 mg/L·h (∼23.5-fold) and versus 42.27 mg/L·h (∼3.1-fold), extend half-life to 10.41 h versus 0.84 h, and reduce infarct size to 28% compared to 51% (∼55% reduction), 37% (∼24% improvement), and 46%. Drug release is prolonged to 60 h versus 48 h and 24 h, demonstrating superior cardioprotection (Zhang et al., 2018; Mao et al., 2018).

## 16 Translational gaps and clinical readiness of nanomedicines

Across the seventeen studies reviewed, a consistent translational gap emerges: while all formulations demonstrate superior cardioprotection compared to free drugs in rodent models, none meet the full criteria required for clinical advancement. Regarding scalability and manufacturing reproducibility, nano-astaxanthin (hot high-pressure homogenization), ferulic acid SLNs (nano-template engineering), TFDM-SLNs (high-shear homogenization), and RGD-puerarin SLNs (solvent evaporation) employ techniques amenable to scale-up, yet batch-to-batch variability data are universally absent. Stability data, reported in only a subset of studies, show nano-astaxanthin stable for 3 months at 4 °C and 25 °C; PNS-HLV stable for 12 months at 4 °C; berberine liposomes with long-term colloidal stability; and CurNisNp with 3-month stability, while the remaining studies omit or provide insufficient stability profiles. Pharmacokinetics and biodistribution are reported in four studies: RGD-puerarin SLNs show 3.3-fold increased AUC, 3.6-fold extended half-life, and 23.1-fold higher myocardial accumulation; RGD-S/P-LPNs demonstrate ∼10- to 11-fold AUC increases; berberine liposomes show preferential infarct accumulation with macrophage co-localization; and TPP-TPGS/TN LPNs achieve 23.5-fold increased AUC with 10.4-h half-life. The remaining 13 studies provide no pharmacokinetic or biodistribution data. Large-animal model validation is absent across all 17 studies, with efficacy confined to rodent models. Long-term safety beyond acute histopathology is unreported, and no formulation addresses manufacturing reproducibility, regulatory pathway considerations, or proposes criteria for clinical advancement. Collectively, while these nanoformulations consistently enhance cardioprotection via improved bioavailability, targeted delivery, and sustained release, translation is currently hindered by absent scalability data, limited stability and pharmacokinetic profiling, lack of large-animal validation, and no defined regulatory strategy—gaps that must be prioritized to enable clinical translation of cardioprotective nanomedicines.

## 17 Conclusion

Ischemic heart disease represents a complex and multifaceted global health crisis, driven by a convergence of genetic predispositions, metabolic dysregulation, lifestyle factors, and environmental exposures. This review summarizes the current understanding of IHD, encompassing its epidemiological burden and diverse risk factors, as well as its intricate pathophysiology, involving endothelial dysfunction, atherosclerosis, and inflammation. Standard management, incorporating lifestyle modifications, comprehensive pharmacological therapy, and advanced revascularization techniques such as PCI and CABG, has significantly improved outcomes. Furthermore, the therapeutic potential of natural bioactive compounds offers promising complementary strategies by targeting key molecular pathways such as oxidative stress and inflammation, however they have not yet been assessed for clinical trials. The future of IHD management is rapidly evolving towards a more personalized and mechanism-driven paradigm. Innovations in precision medicine, targeted anti-inflammatory therapies, regenerative medicine, and modulation of the gut-heart axis are poised to address the significant residual risk that persists despite current optimal care. By integrating these emerging strategies with established treatments, there is potential to transition from managing symptoms to restoring vascular and myocardial health, ultimately reducing the global burden of this pervasive disease. Despite advances in IHD research, the clinical integration of multi-omics data and polygenic risk scores remains limited, necessitating further research to develop a validated model for an individualized prediction strategy and pharmacogenomic-guided therapy. Additionally, the long-term efficacy and safety of emerging treatments, including immunomodulatory agents, regenerative therapies, and gut-microbiome modulators, require further verification through large-scale, long-term clinical investigations.

## Glossary

ABCA1: ATP-binding cassette transporter A1.
ABCG1: ATP-binding cassette subfamily G member 1.
ACC: Acetyl-coa carboxylase.
ACS: Acute coronary syndromes.
ALT: Alanine aminotransferase.
AMI: Acute myocardial infraction.
AMPK: AMP-activated protein kinase.
ANF: Atrial natriuretic factor.
APP: Amyloid precursor protein.
AST: Aspartate aminotransferase.
AUC: Area under the curve.
BACE1: Β-secretase-1.
Bad: Bcl-2–associated agonist of cell death.
Bax: Bcl-2–associated X protein.
BB-lip: Liposome-encapsulated berberine.
Bcl-2: B-cell lymphoma 2.
Bcl-xL: B-cell lymphoma-extra-large.
BMI: Body mass index.
CABG: Coronary artery bypass grafting.
CAD: Coronary artery disease
CAT: Catalase.
CCS: Chronic coronary syndromes
**CD31** ^**+**^: Cluster of differentiation 31^+^.
CFR: Coronary flow reserve.
CHD: Coronary heart disease
CK-MB: Creatine kinase-MB.
COX-2: Cyclooxygenase-2.
CPK: Creatine phosphokinase.
CRP: C-reactive protein.
cTnI: Cardiac troponin I.
CYP7A1: Cytochrome P450 Family 7 Subfamily A Member 1.
DALYs: Disability-adjusted life years.
DL: Drug loading.
DM: Diabetes mellitus.
DPPC: Dipalmitoylphosphatidylcholine.
DSPE-PEG2000: 1,2-Distearoyl-sn-glycero-3-phosphoethanolamine–Poly(ethylene glycol) 2000.
EE: Entrapment efficiency.
EGCG: Epigallocatechin gallate.
eNOS: Endothelial NO synthase.
EPC: Endothelial progenitor cell.
ERK1/2: Extracellular signal–regulated kinase 1 and 2.
ET-1: Endothelin-1.
FAS: Fatty acid synthase.
FA-SLNs: Ferulic acid–loaded solid lipid nanoparticles.
FGF: Fibroblast growth factor.
FXR: Farnesoid-X-receptor.
GBD: Global burden of disease
GDP: Gross domestic product.
GE: Ginger extract.
GLP-1: Glucagon like peptide-1.
GM-CSF: Granulocyte–macrophage colony-stimulating factor.
GPX: Glutathione peroxidase.
GSH: Reduced glutathione.
GST: Glutathione S-transferase.
HDL-c: High density lipoprotein cholesterol.
HO-1: Heme oxygenase-1.
hs-cTn: High-sensitivity cardiac troponin.
ICAM-1: Intercellular adhesion molecule-1
IHD: Ischemic heart disease
IL-1β: Interleukin 1-beta.
IL-6: Interleukin-6
INOCA: Ischemia with non-obstructive coronary arteries.
IκB: Inhibitor of kappa B.
JAK2/STAT3: Janus kinase 2/signal transducer and activator of transcription 3 signaling pathway.
JNK: C-Jun N-terminal kinase.
KIM-1: Kidney injury molecule-1.
LC3B: Microtubule-associated protein 1 light chain 3 beta.
LDH: Lactate dehydrogenase.
LDL-c: Low-density lipoprotein cholesterol.
Lp(a): Lipoprotein(a).
LPO: Lipid peroxidation.
MACE: Major adverse cardiovascular events
MAPK: Mitogen-activated protein kinase.
MCP-1: Monocyte chemoattractant protein-1.
MDA: Malondialdehyde.
MENA: Middle East and North Africa.
MMP-2/9: Matrix metalloproteinases.
MOF: Metal-organic framework.
mRNA: Messenger RNA.
NADPH: Nicotinamide adenine dinucleotide phosphate
nano-ASX: Nano-formulated astaxanthin.
nC: Curcumin nanoparticles.
NF-κB: Nuclear factor kappa B.
NLCs: Nanostructured lipid carriers.
NLRP3: NOD-like receptor pyrin domain-containing 3.
NO: Nitric oxide.
NOX: NADPH oxidase.
NQO1: NAD(P)H quinone oxidoreductase-1.
Nrf2: Nuclear factor erythroid 2–related factor 2.
NR-LGNPs: Lignin-based naringin-loaded nanoparticles.
NSTEMI: Non-ST-elevation myocardial infarction.
OxLDL: Oxidized LDL.
p300-HAT: P300 histone acetyltransferase.
PCI: Percutaneous coronary intervention.
PCSK9: Proprotein convertase subtilisin/kexin type 9.
PDI: Polydispersity index.
PGC-1α: Peroxisome proliferator–activated receptor-gamma coactivator 1-alpha.
PGG: Penta-O-galloyl-β-D-glucopyranose.
**PGI**_**2**_: Prostacyclin.
PI3K/Akt: Phosphoinositide 3-kinase/protein kinase B.
PNS-HLV: Panax notoginsenoside–loaded core–shell hybrid liposomal vesicles.
POD: Peroxidase.
**PPAR**-γ: -γPeroxisome proliferator-activated receptor-γ.
PRS: Polygenic risk scores.
PU: Polyurethane.
RAAS: Renin–angiotensin–aldosterone system
RGD/PEG-PUE-SLN: RGD-modified and pegylated solid lipid nanoparticles encapsulating puerarin.
ROS: Reactive oxygen species.
RR: Risk reduction.
SGLT2: Sodium–glucose cotransporter 2
SIRT1: Sirtuin-1.
SOD: Superoxide dismutase.
STEMI: ST-elevation myocardial infarction.
TA: Tannic acid
TAC: Total antioxidant capacity.
TAG: Triglyceride
TAK1: Transforming growth factor-β-activated kinase 1.
TBARS: Thiobarbituric acid reactive substances.
TF: Tissue factor.
TFDM-SLNs: Total flavonoid extract from Dracocephalum moldavica L.
TGF-β/Smad: Transforming growth factor–beta/smad signaling pathway.
TLR4: Toll-like receptor 4.
TLRs: Toll-like receptors.
TMAO: Trimethylamine N-oxide.
TNF-α: Necrosis factor-alpha
TTC: 2,3,5-triphenyltetrazolium chloride.
ULK1: Unc-51 like autophagy activating kinase 1.
VA: Vanillic acid.
VB: Vitamin B
VB@MOF/TA: Vitamin B-loaded metal–organic framework/tannic acid.
VCAM-1: Vascular cell adhesion molecule-1
VEGF: Vascular endothelial growth factor.
vLDL-c: Very low-density lipoprotein cholesterol.
**α-SMA** ^**+**^: Alpha-smooth muscle Actin.
